# Application of Artificial Intelligence in Aquaculture, Processing, Safety, and Traceability in the Industry of Aquatic Products: A Review

**DOI:** 10.3390/foods15183205

**Published:** 2026-09-10

**Authors:** Jingshu Chen, Zengtao Ji, Chuanheng Sun, Yi Yang, Hongbing Fan, Yueyue Liu, Qian Xu, Ce Shi

**Affiliations:** 1 College of Food Science and Engineering, Tarim University, Alar 843301, China; 2 Production & Construction Group Key Laboratory of Special Agricultural Products Further Processing in Southern Xinjiang, Alar 843301, China; 3 Information Technology Research Center, Beijing Academy of Agriculture and Forestry Sciences, Beijing 100097, China; 4 National Engineering Research Center for Agri-Product Quality and Safety Traceability, Beijing 100097, China; 5 School of Computer and Artificial Intelligence, Beijing Technology and Business University, Beijing 100048, China; 6 Department of Animal and Food Sciences, University of Kentucky, Lexington, KY 40546, USA; 7 Department of Food Science, University of Copenhagen, Rolighedsvej 26, 1958 Frederiksberg, Denmark; 8 School of Food and Health, Beijing Technology and Business University, Beijing 100048, China

**Keywords:** perceptual AI, predictive AI, control AI, generative AI, aquatic products industry chain

## Abstract

The aquatic products industry has experienced rapid development due to the growing global demand for high-quality proteins. However, conventional production models remain constrained by multifaceted impediments, including disease outbreaks, environmental stressors, microbial contamination, and operational inefficiencies in processing, quality control, and logistics. Artificial intelligence (AI) offers targeted methodological solutions to these challenges. From a functional perspective, this review categorizes artificial intelligence into four major types: perception, prediction, control, and generation, and systematically evaluates its application progress in aquaculture, processing, quality inspection, and traceability fields. Perception AI constitutes the data acquisition and digitization layer, utilizing computer vision, sonar, and multimodal fusion technologies to establish digital mappings from environmental parameters to biological indicators. Prediction AI employs machine learning and deep learning algorithms to transform historical datasets into quantitative forecasts regarding water quality dynamics, disease risks, and production trends. Control AI translates decision-making protocols into precise, autonomous regulatory actions for aquaculture environments and processing workflows through fuzzy logic and model-based predictive control. Generation AI leverages large language models and generative adversarial networks to demonstrate innovative capabilities in data augmentation, solution optimization, and virtual simulation. Collectively, these applications optimize core production processes while significantly enhancing product quality, processing efficiency, safety management, and traceability systems. Future research directions will prioritize the development of robust, interdisciplinary AI technologies with superior integration capabilities.

## 1. Introduction

Fish have become an indispensable component of human diets due to their rich content of premium animal proteins and unsaturated fatty acids [1]. According to the UN Food and Agriculture Organization (FAO)’s “State of World Fisheries and Aquaculture 2024” report, global aquatic product production has grown steadily at a compound annual growth rate of 3.2% over the past decade, which indicates that the aquatic products industry is in a stage of rapid development. However, behind this rapid development, aquaculture, processing, safety, and traceability of aquatic products face notable issues arising from the gap between output expansion and high-quality development. Aquaculture faces issues such as high feeding costs, aquatic ecosystem imbalance, low efficiency, weak ability to control diseases, and a shortage of high-quality fry [2,3,4]. Aquatic product processing involves inadequate cutting precision, high waste rates, microbial contamination, low level of industrialization, and lacking unified processing standards [5,6,7]. In aquatic product safety, there are issues such as low detection efficiency, difficulty in quickly identifying contaminants like microorganisms, and insufficient freshness detection and prevention technologies [8]. In terms of traceability, there are problems such as fragmented data across the entire chain and relatively low credibility of traceability information [9]. However, traditional methods to solve the above problems are often time-consuming, non-intelligent, highly destructive, less predictable, and inefficient. In recent years, AI technologies have demonstrated significant advantages in precision, intelligence, efficiency, dynamism, greenness, and collaboration, greatly improving the efficiency of solving these problems.

Since the Dartmouth Conference formally established the discipline of AI in 1956, the field has made a historic leap from laboratory theory to industry application. With the development and improvement of Transformer architecture, large pre-trained models, and exponential GPU computing power, AI has made revolutionary breakthroughs in aquatic industries like aquaculture, processing, safety inspection, and traceability [3,10,11]. Quaade et al. [12] employed remote sensing and computer vision technologies to train a YOLOv5 model for the identification of marine aquaculture cages in aerial and satellite imagery, thereby markedly enhancing the efficiency and accuracy of aquaculture surveys. Zeng et al. [13] integrated near-infrared spectroscopy and hyperspectral imaging with drying equipment, employing neural networks to predict aquatic product drying conditions through a multi-parameter fusion model. Zhang et al. [14] took *Scophthalmus maximus* as the research object, proposed an improved fuzzy proportional–integral–derivative (PID) control system, optimized its parameters, verified it through simulated low-temperature anhydrous live transportation, and found its oxygen control effect was better than traditional algorithms, which is beneficial to the optimization of live fish circulation. Zhao et al. [15] developed a generative AI model that utilized underwater sensor data to calculate the daily dietary requirements of the herbivorous *Ctenopharyngodon idella*, reducing feed waste by 15% while improving growth efficiency. Therefore, when summarizing and classifying AI technologies, the main technologies can be divided into perceptual AI, predictive AI, control AI, and generative AI. Perceptual AI relies on technologies such as computer vision, sonar signals, and sensors to achieve real-time perception and understanding of both the physical world and digital information. Predictive AI uses big data analysis and machine learning algorithms to forecast operational trends and identify potential risks. Control AI uses reinforcement learning and intelligent decision-making algorithms to achieve precise regulation of dynamic systems. Generative AI uses Generative Adversarial Networks (GANs), diffusion models, and large language models (LLMs) to achieve data generation and solution optimization [16].

Currently, most systematic review studies on the application of AI technologies in the aquatic products industry primarily focus on the aquaculture sector; however, there is a lack of reviews that systematically classify AI technologies and their applications, as well as their deployment across various application scenarios—including aquaculture, processing, safety inspection, and product traceability. Therefore, this review first conducted a literature search across the Web of Science, Scopus, PubMed, and IEEE Xplore databases, identifying 167 publications published between 2020 and 2026. Using the following search keywords (AI, machine learning, deep learning, computer vision, neural networks, generative AI, fuzzy logic control, model predictive control, large language models, GAN, aquaculture, livestock and poultry product processing, product freshness, traceability), and after excluding literature that fell outside the scope, lacked direct relevance, contained incomplete information, or lacked substantive technical or validation data, a total of 89 publications were retrieved. Based on these publications, this review classifies AI into four types—perceptual AI, predictive AI, control-oriented AI, and generative AI—and elucidates the underlying principles of each type of AI.

**Figure 1:**
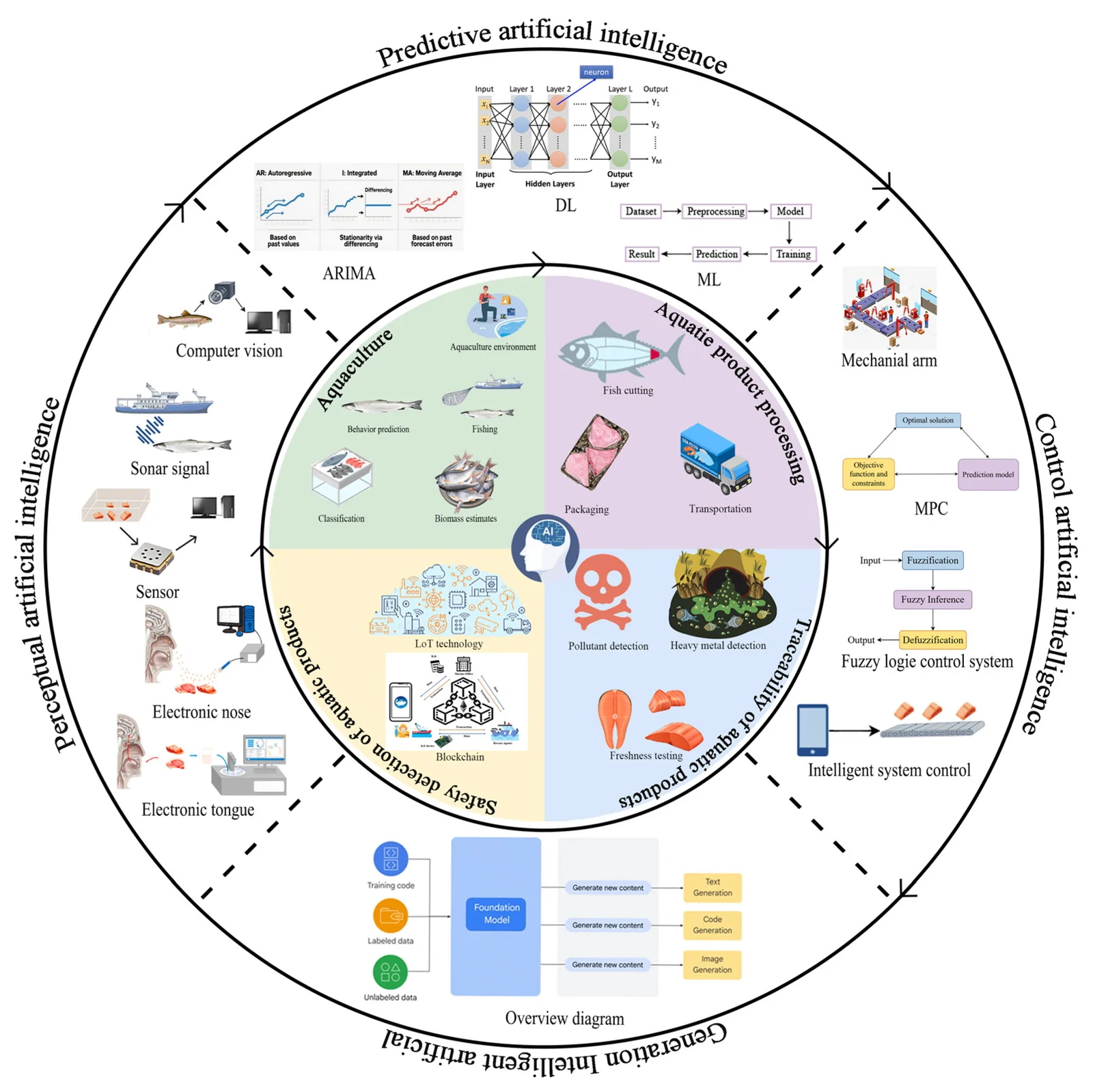
Schematic diagram of the review framework.

For the aquatic products industry, this classification framework enables practitioners to make more informed decisions regarding digital infrastructure investment, thereby accelerating the transition toward precise, resilient, and sustainable production systems. For researchers, this framework offers new perspectives for future empirical studies, facilitates the systematic organization of existing literature, and helps identify underexplored interdisciplinary fields. Ultimately, we hope that this review will not only clarify current challenges and future research directions but also provide insights with practical value (Figure 1).

## 2. AI Technology in the Aquatic Products Industry

Many artificial intelligence technologies are inherently multifunctional, capable of serving multiple categories simultaneously. To ensure consistency and reproducibility in the classification methodology, we have adopted the following principles:

When a technology performs multiple functions within a specific application scenario, it should be classified according to its primary or terminal function within that scenario. For example, computer vision technology used for real-time fish freshness grading on processing lines should be classified as perceptual artificial intelligence—since its primary role is to perform quality assessment by analyzing sensory data, although these same visual features can subsequently be fed into a predictive shelf-life modeling system. Conversely, when computer vision technology is integrated into a feedback loop to adjust feeding rates in real time based on observed fish behavior, this technology should be classified as control-oriented artificial intelligence—because, in this case, its perceptual function serves merely as a subordinate component within an automated decision–execution cycle. The classification of technologies is context-dependent, rather than stemming from the inherent characteristics of the algorithms themselves. For example, a Convolutional Neural Network (CNN) might be classified as perceptual artificial intelligence (e.g., for disease detection based on images) in one study, but as predictive artificial intelligence (e.g., for predicting biomass growth based on time-series derived from images) in another study. Each paper reviewed is classified according to the authors’ specific application approach for the technology, rather than based on the technology’s general functionality.

These four categories correspond to different stages within a workflow: Perceptual AI handles data collection and semantic parsing; Predictive AI leverages time-series or causal reasoning to forecast future states; Control AI transforms the predictive outcomes or perceptual information into automated execution or optimization operations; while Generative AI enhances the functionality of the aforementioned three stages by generating new data, new designs, or knowledge-based outcomes. Technologies spanning multiple stages are classified according to the stage in which they deliver a core value proposition within the specific application scenario being evaluated.

### 2.1. Perceptual AI

**Figure 2:**
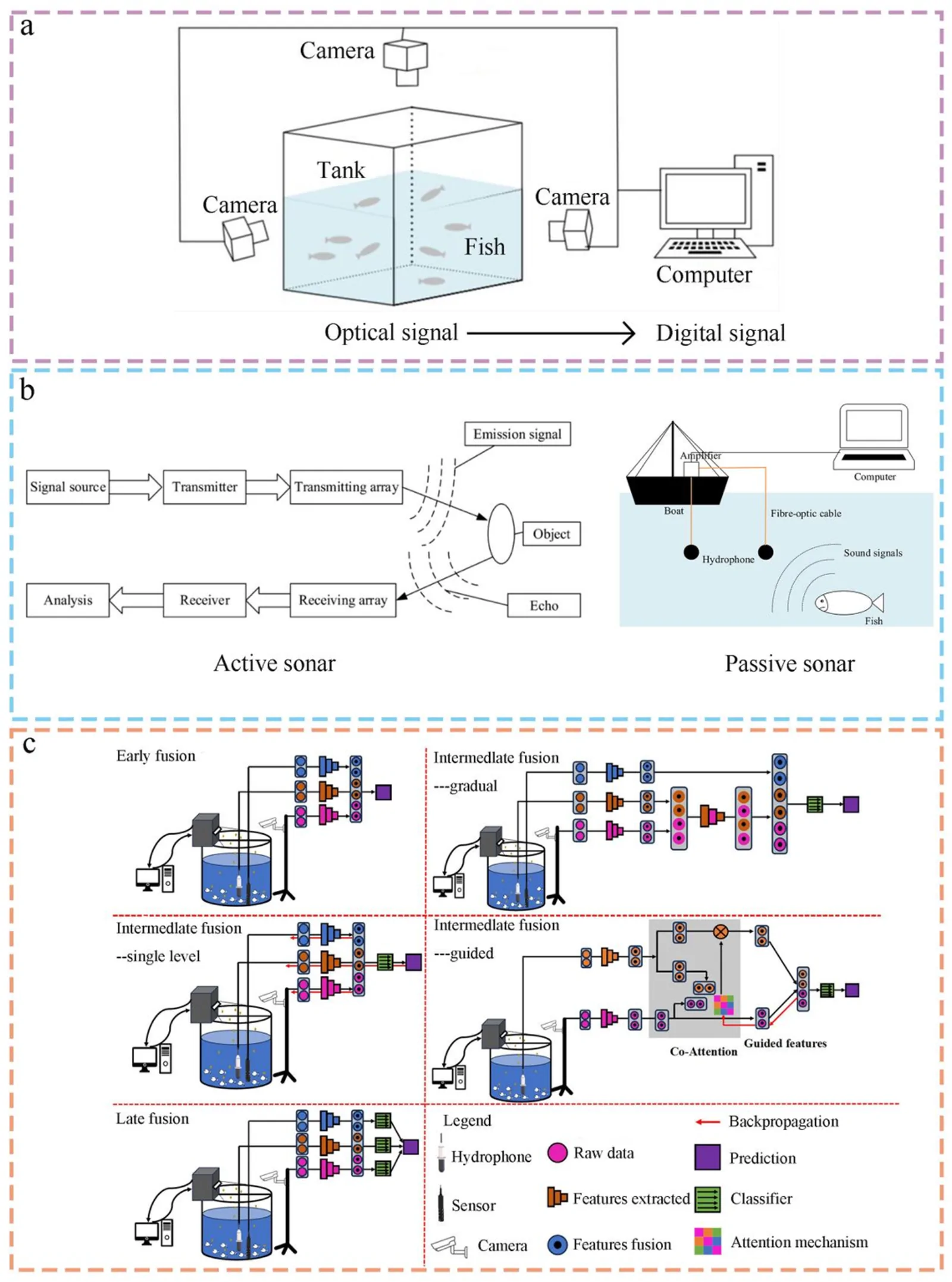
(**a**) Computer vision technology workflow picture. (**b**) Sonar technology workflow picture. (**c**) Multimodal data fusion workflow picture. Adapted with permission from Ref. [18]. Copyright 2024, Elsevier.

Perceptual AI is crucial in the aquatic products industry, which can comprehensively and in real time monitor the aquaculture environment, biological signs, and equipment status through computer vision, sonar signals, and sensors. As the foundation of the AI’s closed loop, perceptual AI enables machines to gather environmental data, recognize features, and respond, much like human senses such as vision, smell, touch, and hearing [17]. This technology converts various signals (such as images, sounds, and environmental data) into digital signals (Figure 2).

#### 2.1.1. Computer Vision Technology

In recent years, computer vision technology has been widely used in aquatic product species identification, counting, behavior analysis, and other aspects [12,19,20]. Computer vision, a key area of perceptual AI, involves acquiring image or video data using visual sensors like cameras. It preprocesses these data using techniques such as denoising and distortion correction, and then applies deep learning to extract features for tasks including image classification, target detection, recognition, segmentation, and tracking, converting digital data into comprehensible information [21]. Hu et al. [22] used overhead cameras combined with multi-class support vector machines (MSVMs) to classify fish types according to color and texture features. However, due to the manual addition of images, classification errors still occurred. Atienza-Vanacloig et al. [23] proposed a variability-adaptive 2D model. By using computer vision technology, this model can identify tuna from a variety of fish species, achieving a success rate as high as 90.6%. Reference [24] proposed a method for the systematic detection, localization, and recognition of local abnormal behaviors. Based on an improved motion influence map and Recurrent Neural Network (RNN), this method demonstrates superiority over many other advanced approaches.

#### 2.1.2. Sonar Technology

As the foundation of computer vision technology, lighting technology is affected by water density, light, and turbidity in practical applications, so sonar technology becomes a more effective information collection tool. Sonar achieves target detection, environmental perception, and aquatic behavior analysis by transmitting and receiving acoustic signals, integrating acoustics, signal processing, and intelligent algorithms [25]. The sonar system works by converting electrical signals into acoustic energy, amplifying echo signals, performing analog-to-digital conversion, and using algorithms such as the fast Fourier transform and matching filter to extract target features [26]. Sonar systems can be divided into active sonar and passive sonar. Active sonar intermittently transmits acoustic waves and acquires data regarding the target’s location and range by analyzing the received reflected echoes [27]. The main active sonar systems used in aquaculture are Multi-Beam Echo Sounder (MBES) systems, sonar imaging systems, and acoustic telemetry systems. Passive sonar enables specific research by receiving and processing radiated noise from underwater targets, and it is composed of a single hydrophone detection system. The only passive sonar application in aquaculture is the hydrophone monitoring system [28]. The main applications of sonar systems in aquatic product aquaculture are biomass statistics and morphological analysis, behavior analysis, and environmental management. Hassan et al. [29] utilized acoustic telemetry tags and a LoRa LPWAN network to form a fish internet of things (loF), enabling real-time collection of behavioral data such as the swimming trajectories and depths of salmon. The 4 sets of LoRa Add-on Modules (LAM) and acoustic receivers they deployed form a distributed monitoring network within the range of 150 m to 2.5 km, which can effectively detect fish behaviors. Mallekh et al. [30] used acoustic sensors to monitor the sounds emitted by fish during feeding, and after data processing by acoustic receivers, they could estimate the fish’s demand for feed pellets. However, it is still affected by environmental factors such as dissolved oxygen in the water on the receiving frequency.

#### 2.1.3. Multimodal Data Fusion

Computer vision and sonar technologies are popular single-modal methods with advantages over physical sensors like temperature and pH sensors. However, they face challenges such as image interference and data processing limitations. Due to the complexity of the aquatic products industry, single-modal technologies fall short in capturing all necessary information [31]. Conversely, multi-modal data fusion synthesizes information from diverse sources, offering a holistic understanding and substantial potential for advancing the aquatic products industry.

Multimodal data fusion technology achieves more efficient information extraction and analysis by integrating diverse modal data such as visual, acoustic, spectral, remote sensing, and blockchain data [32,33,34]. The early fusion methods in multimodal technologies were mainly reflected at the data level. In the early stage, researchers preprocessed and aligned data from different modalities and then adopted traditional machine learning methods for feature extraction and classification. While this method is relatively straightforward and can effectively integrate multimodal data to a certain degree, it encounters problems related to data loss and mismatch when applied to complex remote sensing images [35,36,37]. As deep learning techniques have advanced, researchers have increasingly focused on feature-level fusion in the context of multimodal integration. These approaches enable more thorough excavation of inter-modal complementary information and boost the effectiveness of semantic segmentation by fusing feature representations from diverse modalities. Such methods are classified into early fusion, intermediate fusion, and late fusion according to fusion timing [38]. Early fusion: Prior to the multimodal data entering the encoder, feature fusion is conducted. Although early fusion methods are simple, they may lead to feature conflicts and information loss between different modal data. This phenomenon arises from the distinct statistical characteristics inherent to data of varying modalities. Direct concatenation or weighted averaging may inadequately account for these inter-modal differences, potentially leading to information redundancy or inconsistency, which, in turn, adversely impacts segmentation performance [39]. Intermediate fusion: This approach conducts feature fusion at the intermediate stage of feature extraction, systematically integrating multimodal data features across various layers of the model while maintaining the distinct attributes of each modality. By incrementally reconciling inter-modal differences through a layer-by-layer fusion process, it augments the representational capacity of multimodal data. The strategy of performing fusion at the intermediate layer and progressively integrating multimodal data features from different model layers has gained widespread preference in contemporary research. Furthermore, intermediate fusion can be classified into three distinct categories: single-level fusion, progressive fusion, and guided fusion. In single-level fusion, features from multiple modalities are integrated at a single stage after being extracted by their respective encoders. Conversely, progressive fusion involves the gradual integration of features across multiple levels, thereby allowing the model to capture inter-modal relationships at varying levels of abstraction. Guided fusion, on the other hand, incorporates a guidance mechanism that utilizes a common-attention mechanism to refine and optimize the fusion process of multimodal features [40]. Late fusion: In the latter stage of the feature extraction process, this procedure is typically conducted following the independent processing of each modality’s features by the decoder, prior to their integration. Although this approach maintains the unique characteristics of each modality and reduces information loss, it may not fully leverage the complementary information present between modalities. In particular, during the encoding phase, late fusion is constrained in its capacity to effectively capture the spatial relationships among different modalities, which can subsequently negatively affect segmentation results [41]. In recent years, alongside feature-level fusion, a novel approach called model-level fusion has emerged [18]. As a broader integration concept, model-level fusion encompasses three distinct phases: early, mid, and late fusion stages, and has become a standard strategy in the machine learning and deep learning industry. Its core objective lies in achieving knowledge expansion through joint learning of multimodal features, enabling both single-modal feature extraction and splicing [42]. However, this approach has seen limited application in aquatic product domains.

In the aquatic products industry, the technology of multimodal fusion can overcome the limitations of single-modal technologies. By combining data from different sources, it forms a more comprehensive and accurate dataset. The fusion of such multi-source data not only improves the quality and reliability of the data but also provides a richer information foundation for intelligent management in the aquatic products industry. Gu et al. [43] proposed a novel Multimodal Fusion Interaction Network (MMFINet), which enhances the accuracy and robustness of feeding intensity analysis by integrating audio, video, and dissolved oxygen data. Zhang et al. [44] developed a microenvironmental and respiratory angle sensor system to non-invasively assess the respiratory stress state of sturgeon subjected to waterless and low-temperature conditions. They effectively realized a comprehensive evaluation of respiratory stress states by predicting respiratory efficiency signals using models such as Linear Regression (LR), Back Propagation Neural Network (BPNN), Support Vector Regression (SVR), and Radial Basis Function Neural Network (RBFNN). In conclusion, multimodal perception fusion breaks down the data barriers between diverse modalities such as vision, acoustics, spectroscopy, remote sensing, and blockchain. It drives the aquaculture industry towards in-depth transformation in the direction of digitalization, intellectualization, and transparency, achieving multiple improvements in yield, quality, and sustainability. Consequently, multimodal technology exhibits significant potential for development and application within the aquatic products industry.

### 2.2. Predictive AI

**Figure 3:**
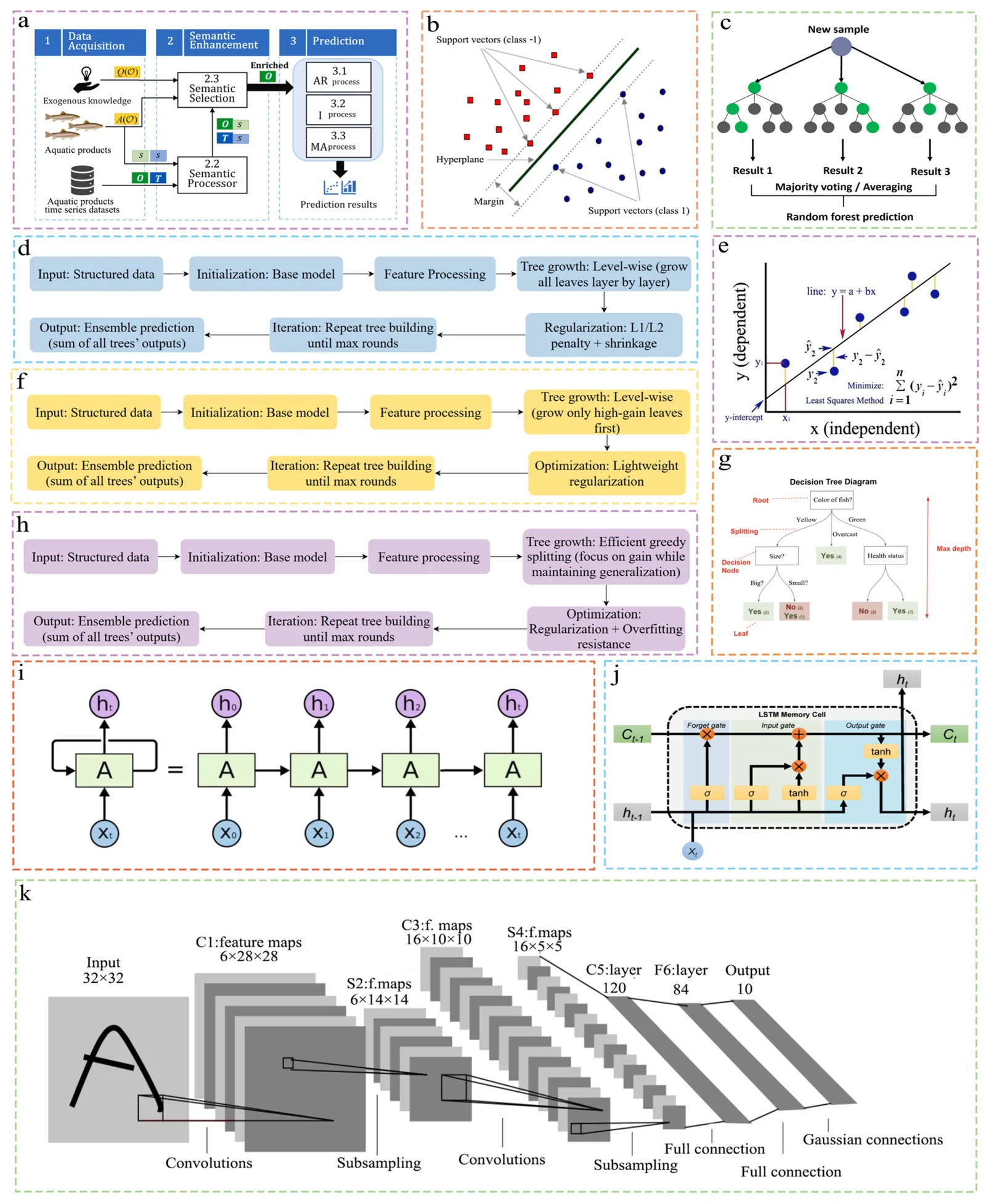
(**a**) The architecture of the ARIMA model. Q: Query external knowledge. O: Object. A: Attribute. S: State. T: Time. AR: Auto-Regressive. I: Interpolation. MA: Moving Average. (**b**) The architecture of the SVM model. (**c**) The architecture of the Random Forest model. (**d**) The architecture of the XGBoost model. (**e**) The architecture of the Linear Regression model. y_i_: The true value of the i-th sample. ŷ_i_: The predicted value for the i-th sample. y_i_-ŷ_i_: Residual. (**f**) The architecture of the LightGBM model. (**g**) The architecture of the Decision Tree model. (**h**) The architecture of the CatBoost model. (**i**) The architecture of the RNN model. x_0_, x_1_,…, x_t_: Input vectors for different time steps. h_0_, h_1_,…, h_t_: Hidden layer states at different time steps. A: Neural network module. (**j**) The architecture of the LSTM model. x_t_: Input for the current time step. h_t-1_: The hidden state from the previous time step (output). C_t-1_: State from the previous time step (Long-term memory). C_t_: The state after the current time-step update. h_t_: Hidden state at the current time step (output). σ: Sigmoid activation function. tanh: Hyperbolic tangent activation function. (**k**) The architecture of the CNN model.

Predictive AI technologies leverage machine learning and other core methodologies to forecast and analyze key indicators in the aquatic products industry (Figure 3). This enables real-time optimization of production processes and predictive quality control, ultimately enhancing production efficiency and product quality. The application of this technology not only helps to enhance the safety and sustainability of aquatic products but also provides a solid technical foundation for control AI [45].

#### 2.2.1.  Traditional Time Series Models

The Auto-Regressive Integrated Moving Average (ARIMA) model was a predominant method in early aquatic product forecasting [46]. This univariate stochastic model integrates autoregressive (AR), differencing (I), and moving average (MA) components to effectively model non-stationary time series characterized by trends and autocorrelation. Mathematically, the ARIMA (p, d, q) model is represented as follows:

$$\left(1-\sum \varphi_{i}L^{i}\right){\left(1-L\right)}^{d}X_{t\;}=\;c\;+\left(1+\sum \theta_{j}L^{j}\right)\varepsilon_{t}$$

where X_t_ denotes the observed value at time t; L is the lag operator; φ_i_ and θ_j_ represent the model coefficients; and ε_t_ is the white noise error term. The differencing term ensures stationarity prior to the application of AR and MA terms [47].

#### 2.2.2. Machine Learning Models

Machine learning models (ML) such as XGBoost, LightGBM, Random Forest, CatBoost, Support Vector Machine (SVM), Decision Tree, and Linear Regression are widely used in the field of the aquatic products industry (Table 1). They exhibit excellent performance, especially when dealing with large-scale datasets and complex problems. The popularity of these models is not only due to their prediction accuracy but also to their flexibility and adaptability across different application scenarios.

(1) XGBoost is an optimized ensemble model based on Gradient Boosting Decision Trees (GBDT). Its core mechanism not only iteratively optimizes the loss function through gradient descent but also incorporates L1 and L2 regularization (applied to leaf node weights and model complexity, respectively) to effectively prevent overfitting. The model supports automatic handling of missing values without manual intervention and enables parallel feature computation through column sampling, significantly enhancing training efficiency [48]. In aquaculture, the model is widely used to predict the biomass of aquatic products [49].

(2) LightGBM is a lightweight gradient-boosting tree model that innovates through histogram optimization (discretizing continuous features into histogram bins) to reduce data storage and computational costs. It replaces the traditional level-wise (tiered) decision tree construction with a leaf-wise (leaf node priority) strategy, prioritizing splits at high-gain leaf nodes to significantly enhance training speed while maintaining accuracy [50]. Siddique et al. [51] analyzed environmental parameters, including pH, ammonia nitrogen, and nitrate levels, using LightGBM to predict fish length and weight.

(3) Random Forest (RF) is an ensemble learning approach employed for supervised classification and regression tasks, which operates by constructing a multitude of decision trees based on the relationships between independent and dependent variables. This method is renowned for its accuracy and its capability to effectively handle small sample sizes as well as high-dimensional feature spaces [52,53]. Saville et al. [54] utilized a random forest model to predict fish mortality risk across five distinct risk levels, attaining an overall accuracy of 78.6% and exceeding 70% accuracy for each individual level.

(4) CatBoost is a gradient boosting tree model specifically optimized for categorical features. Its core advantage lies in eliminating the need for manual encoding of categorical features, as it automatically converts them into numerical variables and learns their relationships with the target variable. By combining categorical features with the target variable for ranking, it generates new numerical features that enhance the model’s generalization ability. Additionally, it supports adaptive learning rates to reduce the workload of manual parameter tuning [55].

(5) The core principle of the SVM is to identify the hyperplane with the maximum margin in high-dimensional space to separate different categories of samples. For datasets that are not linearly separable, Support Vector Machines (SVMs) address this challenge by transforming the problem into a linearly separable one. This is achieved by mapping the data into a higher-dimensional space through the application of a kernel function. The model’s complexity depends solely on the number of support vectors, making it particularly effective for small-sample learning and resistant to overfitting [56]. In aquaculture, the model is mainly used to predict growth, environmental changes, and product freshness.

(6) Decision Tree is a supervised learning model based on a tree structure. Its core logic involves recursively splitting data into subsets using splitting criteria, where each internal node represents a feature judgment, and each leaf node corresponds to a prediction result. The model is highly interpretable, directly generating visual decision rules without requiring complex data preprocessing, while also supporting overfitting reduction through pruning [57]. In the field of aquatic products, it is often used to predict the breeding environment [58,59].

(7) Linear Regression is a linear modeling technique that employs the least squares method to establish a linear relationship between independent and dependent variables. It operates by minimizing the sum of squared errors between observed and predicted values. The model assumes no multicollinearity among variables and that errors follow a normal distribution. It is computationally efficient and highly interpretable, allowing direct assessment of independent variables’ influence on the dependent variable through weight magnitude [60]. Gkikas et al. [61] employed a linear regression model to forecast fish mortality rates, with the scatter plot demonstrating strong predictive accuracy for median values.

#### 2.2.3. Deep Learning Models

With the rapid advancement of deep learning methods, the model’s predictive capabilities have achieved remarkable progress. This breakthrough primarily stems from deep learning’s powerful data representation capabilities and its ability to automatically learn features. Studies have shown that Recurrent Neural Networks (RNNs), long short-term memory networks (LSTMs), and transformers demonstrate exceptional performance in time series prediction tasks, as they can automatically capture temporal dependencies in time series data, thereby improving prediction accuracy [62].

(1) Recurrent Neural Networks (RNNs) are adept at capturing temporal dependencies and dynamic variations in time series data due to their intrinsic cyclic connection architecture. This capability facilitates the precise modeling and prediction of both short-term fluctuations and long-term trends [63], and it has been widely applied in the aquaculture industry within the aquatic products industry [64].

(2) However, as the number of iterations increases, Recurrent Neural Network (RNN) models are susceptible to the phenomenon of gradient explosion. Long Short-Term Memory (LSTM) networks and Gated Recurrent Units (GRUs) represent sophisticated advancements of RNN models, specifically engineered to mitigate the gradient explosion problem that arises during prolonged RNN iterations. Dewi et al. [65] Effectively improved the learning efficiency of the model and reduced aquaculture costs by combining the temporal dependency modeling capability of LSTM-RNN with the advantages of Random Forest models in feature selection and nonlinear data processing.

(3) Although RNN and LSTM models can effectively predict parameters in aquaculture and processing, RNNs may suffer from gradient explosion, and LSTMs may be prone to overfitting [66]. However, Transformers, with their self-attention mechanism, outperform these two models in capturing long-range dependencies. This mechanism enables it to model relationships within the entire input sequence without being limited by a fixed-size receptive field or sequential processing. Zheng et al. [67] introduced a Spatiotemporal Attention Network (STAN) designed to discern the behavioral states of golden pompano schools within aquaculture containers. The model is composed of two primary components: an intuitive attention channel and a perceptual attention channel network. These channels independently extract features, which are subsequently integrated to capture spatiotemporal information. The integrated data is then classified into various fish feeding behaviors and states through a fusion classification channel utilizing the Long Short-Term Memory (LSTM) mechanism. Experimental results demonstrate that STAN achieves a remarkable accuracy rate of 97.97% in identifying feeding behaviors, thereby offering valuable real-time feedback for intelligent feeding systems.

However, predictive AI faces a frequently overlooked issue: the vast majority of model evaluations rely solely on accuracy or R^2^ metrics derived from a single dataset, without accounting for temporal distribution drift (e.g., seasonal variations or climatic anomalies) or spatial domain adaptation (e.g., across different farms or species). The strong performance observed on the training dataset often declines sharply when applied to data spanning multiple years or regions; this fragility in generalization capability severely undermines the reliability of such models as decision-support tools. Furthermore, the “black-box” nature of deep learning models makes it difficult for farmers and regulators to trust their predictive outcomes [68], while the applicability of current explainability methods (such as SHAP and LIME) in the aquaculture sector has not yet been fully validated.

### 2.3. Control AI

**Figure 4:**
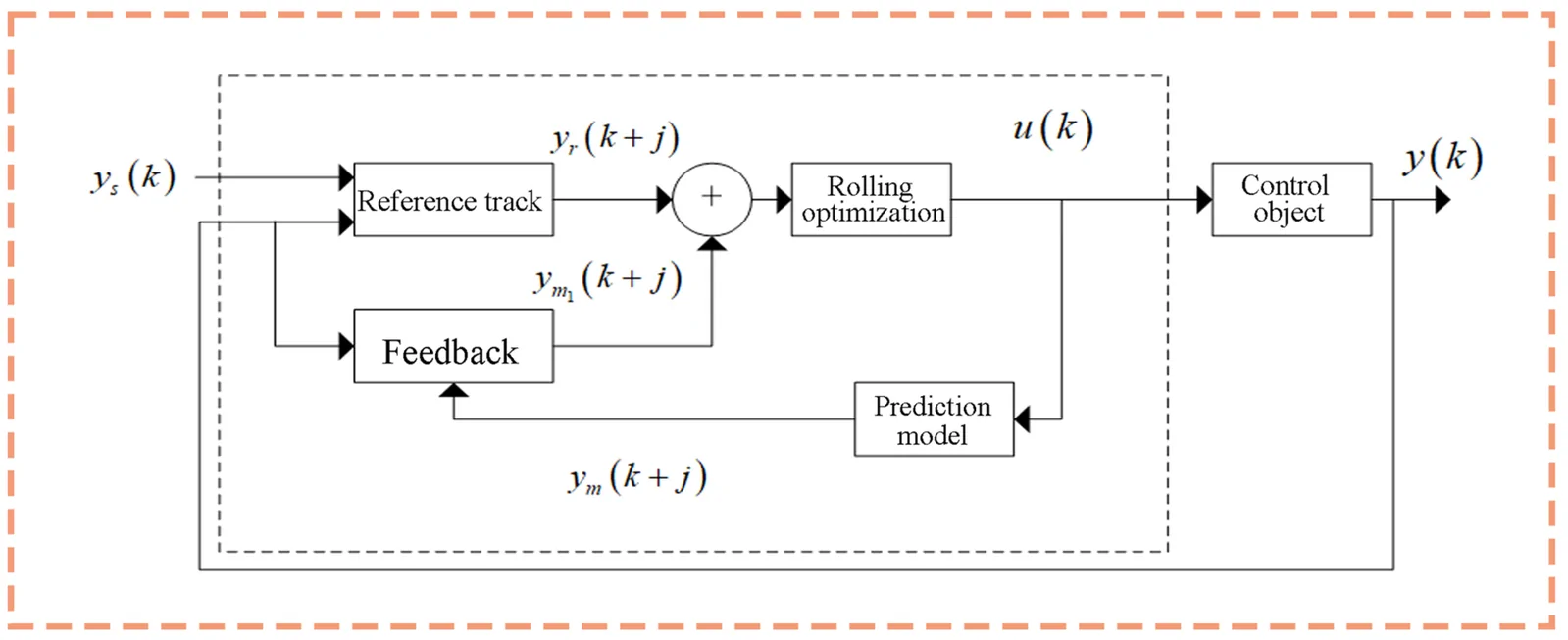
MPC system schematic.

With the deep integration of artificial intelligence and the aquatic products industry, control AI, as the core executive layer, plays a significant role in the precise regulation of aquaculture, processing, transportation, and quality and safety [20,69,70]. To address the complexity of the aquatic products industry, control AI technology is evolving from single-device automation to comprehensive optimization and autonomous decision-making, spanning the entire industrial chain. To address this complexity, control AI in aquaculture has evolved along three distinct paradigms that should be clearly distinguished: (1) Classical control, represented by conventional PID and rule-based manual strategies that rely on fixed mathematical models or empirical thresholds; (2) Intelligent control, typified by fuzzy logic systems that encode expert knowledge into adaptive rule bases to handle uncertainty without requiring precise system identification; and (3) Learning-based control, encompassing model predictive control (MPC), reinforcement learning, and data-driven optimization approaches that iteratively refine control policies through interaction with dynamic environments or predictive models. This section examines these three paradigms in sequence, highlighting their respective strengths, limitations, and application contexts within the aquatic products industry. This section primarily discusses traditional control methods, fuzzy logic systems, and model predictive control (Figure 4).

#### 2.3.1. Traditional Control Methods

Control is critical in the management of aquaculture, processing, transportation, and safety monitoring. Traditional manual control offers flexibility and enables real-time adjustments. For example, workers manually monitor water coloration to replace water, adjust feed quantities based on fish feeding patterns, perform cutting, scrubbing, and packaging procedures, periodically re-ice or refresh water for chilled products during transit, and conduct safety assessments of physicochemical parameters. However, these manual operations suffer from low efficiency due to human limitations and time constraints, heavy reliance on experience, high labor costs, inadequate safety protocols, and lack of standardized procedures. Therefore, how to improve the safety and quality of aquatic products while improving the control efficiency of aquatic products has become an urgent matter. While the traditional control system exhibits certain limitations, it provides a robust foundation for the advancement of fuzzy logic systems, model predictive control, and intelligent control systems.

#### 2.3.2. Fuzzy Logic Control System

The fuzzy logic control system is an advanced control methodology that employs a flexible fuzzy rule base—a framework that aligns more closely with human intuition than with the specialized expertise possessed by experienced process engineers. The core strength of this approach lies in its adaptability and flexibility, enabling effective operation in environments characterized by uncertainty and complexity. Through fuzzy logic systems, control strategies can be dynamically adjusted and optimized without depending on precise mathematical models, thereby achieving efficient management of complex systems [71,72]. However, the performance of traditional FLCs depends heavily on the quality of the predefined rule base and the membership functions; these rules and functions are typically established based on expert knowledge rather than data-driven learning methods.

**Table 1:** Predictive models in machine learning and deep learning.

	Algorithm	Purpose	Software	Sample Size			Preprocess	Performance	References
				Total	Training Set	Testing Set			
ML	XGBoost	Use the XGBoost algorithm to predict the deltamethrin content in aquatic products.	Python 3.9.7	835	584	251	Calculate fluorescence ratio (I_526_/I_450_ + I_630_)	The accuracy rate reaches 95.7%, and the standard deviation is lower than 5.5%.	[48]
	XGBoost	Predicting the weight of Red *tilapia* in river cage culture using XGBoost	Python 3.10	3600	2880	720	Image cropping (2 × 2 m), grayscale conversion, size standardization (64 × 64 pixels), pixel normalization (0–1)	The accuracy value is approximately 76.0 ± 1.9%, and precision is 76.2 ± 1.9%.	[49]
	XGBoost	Predict the gender of *Rainbow* trout using XGBoost.	Python 3.10	1362	/	/	Missing value conversion	The accuracy rate reaches 98% in actual tests.	[73]
	SVM	Predict the concentrations of Co, Cr, Cu, Ni, and Ti in aquatic products using Support Vector Machines.	MATLAB R2016a	700	560	140	data normalization	The R values are all greater than 0.8.	[74]
	SVM	Predict hypoxanthine and detect fish freshness using Support Vector Machines.	Python 3.8	625	500	125	Image cropping (2 × 2 m), grayscale conversion, pixel normalization (0–1)	The prediction coefficient is 94.6% with a standard deviation of 3.79	[75]
	Random Forest	Predict the bioconcentration factor of fish using Random Forest.	/	966	774	192	Molecular descriptor filtering (remove infinite/missing values, low variance, high correlation), RF-RFE feature selection	The R value for training and testing reaches 0.949 and 0.935, respectively.	[52]
	Random Forest	Predict toxin-producing harmful algal blooms (HABs) and fish-killing HABs using Random Forest	Python 3.10	/	80%	20%	Missing data imputation (mean), data standardization (−1 to 1)	The prediction accuracies are 96.1% and 97.8%, respectively.	[76]
	Random Forest, SVM	Estimate the quality of *Sea bass* using Random Forest and Support Vector Machine, respectively.	MATLAB 8.0	295	221	74	Image calibration, segmentation, alignment (ellipse fitting + nearest-neighbor interpolation), feature extraction	The R^2^ of Random Forest is 0.84, and the RMSE is 0.12; those of Support Vector Machine are 0.72 and 0.16, respectively.	[77]
	Decision Tree, Linear Regression, Random Forest	Predict fish mortality rate	Apache Spark 3.40, Apache Cassandra 4.1.0, Spark Connector, Python 3.10, Oracle VM VirtualBox 7.0, Ubuntu Linux 22.04	/	70%	30%	Missing value processing, normalization, and outlier removal	It was found that the Random Forest performed better, with RMSE, MSE, and R^2^ being 0.264, 6.985, and 0.63, respectively.	[61]
DL	1D CNN	Predicting volatile basic nitrogen and total survival number of crayfish	MATLAB 2022	140	105	35	SNV, WTD	R_p_^2^ are 0.9397 and 0.9318, respectively, and RPD are 2.8279 and 2.7560, respectively.	[78]
	LSTM	conduct dynamic fish mortality rate prediction	Python 3.10	100	50	50	Downsampling, interpolation, dummy variable coding, normalization	R^2^ is 0.81, and the RMSE is 0.30.	[79]
	BPNN	Predicting fish feed intake using a backpropagation neural network optimized by a thought evolution algorithm.	MATLAB 8.0	180	60	120	Normalization	The MSE, MAE, and MAPE are 47.53, 6.17, and 0.04, respectively.	[80]
	BPNN	Using a backpropagation neural network to estimate the mass of *Crucian carp*.	Python 3.5	455	/	/	Zoom, grab, cut, grayscale, enhance, binarize	The MAE, RMSE, and R^2^ are 0.0104, 0.0137, and 0.9021, respectively.	[81]
	RNN, LSTM, GRU	Compare the dissolved oxygen predictions of three models to identify the optimal model.	/	5190	5140	150	Outliers are corrected using the median of the adjacent 6 data points, and missing values are replaced with the average of the adjacent 10 data points.	The GRU performs the best in terms of MAE, MSE, MAPE, and R^2^, which are 0.450 mg/L, 0.411, 0.054, and 0.994, respectively.	[82]
	ATCN	Combining the ATCN with the Transformer encoder to predict dissolved oxygen and pH values.	Python 3.10	50,000	10%	10%	Mean ± 3σ outlier detection, time series interpolation to fill missing values, Min-Max normalization	Compared with the standalone Transformer, the MAE and RMSE of this model are improved by approximately 0.375 and 0.363, respectively.	[83]

ML: Machine Learning; DL: Deep Learning; XGBoost: Extreme Gradient Boosting; SVM: Support Vector Machine; RF-RFE: Random Forest - Recursive Feature Elimination; 1D CNN: One-dimensional Convolutional Neural Network; LSTM: Long Short-Term Memory; BPNN: Backpropagation Neural Network; RNN: Recurrent Neural Network; GRU: Gated Recurrent Unit; ATCN: Adaptive Temporal Convolutional Network; RPD: Ratio of Performance to Deviation; RMSE: Root Mean Square Error; MSE: Mean Square Error; MAE: Mean Absolute Error; MAPE: Mean Absolute Percentage Error; SNV: Standard Normalized Variables; WTD: Wavelet Threshold Denoising; HABs: Harmful Algal Blooms.

#### 2.3.3. Model Predictive Control

Model Predictive Control (MPC) is an advanced control strategy. By utilizing dynamic models, MPC predicts future environmental behavior and optimizes control measures accordingly (Figure 4). The fundamental principle of Model Predictive Control (MPC) entails resolving an optimization problem at each sampling instance, taking into account the current state of the system, the desired reference trajectory, and the constraints on both input and output variables. By continuously updating control actions based on real-time measurements and predictive models, MPC effectively adapts to dynamic conditions and disturbances, thereby ensuring optimal performance [84]. Kamali et al. [85] proposed an integrated closed-loop control framework combining Nonlinear Model Predictive Control (NMPC) with Moving Horizon Estimation (MHE). By estimating unobservable states through MHE and optimizing control actions via NMPC, this approach maintains water quality parameters (oxygen, ammonia, nitrate) within set ranges under scenarios including full/state-accessibility limitations, partial state accessibility, aerator failures, and sensor malfunctions. Although control accuracy slightly decreases during partial state access and requires backup equipment for unexpected failures, this solution provides a model-driven advanced control strategy for efficient and stable operation in recirculating aquaculture systems.

Current validation methods for controlled AI systems are predominantly based on simulation environments or short-term experimental campaigns; there is a significant lack of robustness testing under non-ideal operating conditions commonly encountered in real-world aquaculture practices—such as sensor drift, actuator failures, or abrupt changes in water quality. In particular, Model Predictive Control (MPC) relies on precise system dynamic models; however, the bio-physicochemical coupling processes in aquaculture systems exhibit high nonlinearity and time-varying characteristics. As a result, control deviations arising from model mismatch can be more severe than those associated with traditional PID control; this issue is rarely discussed in the current literature.

### 2.4. Generative AI

**Figure 5:**
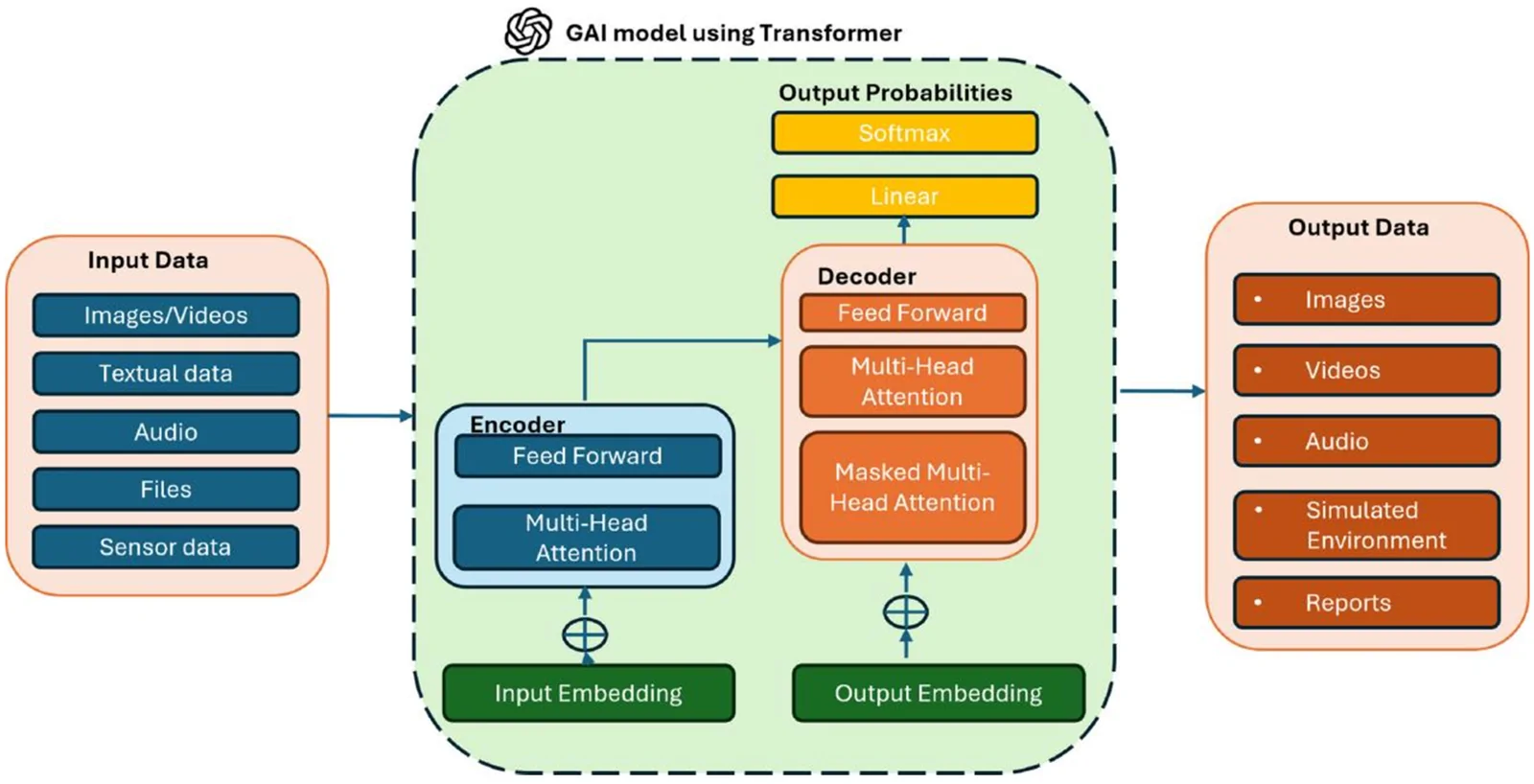
Schematic diagram of generative AI principles. Adapted with permission from Ref. [88]. Copyright 2025, Elsevier.

With advances in computing power, training algorithms, and large-scale data sets, generative AI is making breakthroughs across various industries. Generative AI includes AI models that generate text, images, audio, and video, which are widely referred to as “multimodal generative AI systems (Figure 5)” [86]. The essence of generative AI is to simulate the creative logic in human cognition through algorithms. After training based on massive data, it can independently generate text, images, videos, code, and even 3D models. Different from traditional AI, generative AI has the unique ability to generate new content, designs, and solutions independently [87]. In the aquatic products sector, four primary generative architectures have been explored to date: Generative Adversarial Networks (GANs), Variational Autoencoders (VAEs), Diffusion Models, and Transformers, Large Language Models, and RLHF. Each of these architectures features unique training mechanisms and application domains.

#### 2.4.1. Generative Adversarial Networks

GANs consist of two neural networks—a generator and a discriminator—trained through an adversarial minimax game. The generator uses random noise to synthesize candidate samples, while the discriminator is tasked with distinguishing between real samples and generated samples. Through this iterative adversarial process, the generator learns to produce increasingly realistic outputs without requiring explicit density estimation. In the field of aquatic product research, GANs have been employed to expand limited fish disease image datasets, synthesize realistic underwater scenes for training perception models, and generate synthetic spectral data for freshness detection when experimental data are scarce [89]. Their primary limitation lies in the insufficient diversity of samples generated by the generator during training.

#### 2.4.2. Variational Autoencoders

VAEs adopt a probabilistic latent variable framework, encoding input data into a continuous latent space and decoding sampled latent vectors back into data space. Training maximizes a variational lower bound comprising reconstruction fidelity and latent regularization terms, ensuring the latent space is smooth and continuously sampleable. Compared to GANs, VAEs offer more stable training and explicit density modeling, though generated samples tend to be blurrier. In the aquatic domain, VAEs have been explored for generating plausible molecular structures of bioactive peptides derived from marine by-products and for anomaly detection in processing line sensor data by learning the distribution of normal operating conditions [90].

#### 2.4.3. Diffusion Models

Diffusion models operate through a forward noising process that gradually corrupts data with Gaussian noise and a learned reverse denoising process that recovers data from pure noise. Training optimizes a score-matching objective at multiple noise levels, enabling high-fidelity generation through iterative refinement. Diffusion models have largely superseded GANs in image synthesis quality and training stability. Emerging applications in aquatic products include generating high-resolution histopathology images for parasite detection and creating photorealistic product packaging designs conditioned on textual specifications [91]. Their primary drawback is slow inference due to the multi-step denoising process.

#### 2.4.4. Transformers, Large Language Models, and RLHF

Transformer-based LLMs are pretrained on massive text corpora via next-token prediction, acquiring broad linguistic and world knowledge. This pretraining phase is entirely unsupervised and does not involve reinforcement learning. However, raw pretrained LLMs may produce outputs misaligned with domain requirements. Reinforcement Learning from Human Feedback (RLHF) is a post-training alignment technique specific to LLMs: human evaluators rank model outputs, a reward model is trained on these preferences, and proximal policy optimization (PPO) fine-tunes the LLM to maximize the learned reward. RLHF is therefore not a prerequisite for generative AI broadly, but rather a specialized alignment stage for LLMs [87]. In aquatic products, LLMs have been applied to automated literature mining for aquaculture best practices, multilingual traceability label generation, and conversational decision support for small-scale farmers.

Overall, the application of generative AI in the aquaculture sector is still in the proof-of-concept phase; its most fundamental limitation lies in the inability to guarantee the factual accuracy and reliability of the generated content [92]. Additionally, generative models are highly dependent on the distribution of their training data; when the range of aquaculture scenarios covered by the training data is limited, the diversity of the generated content may instead amplify biases rather than fill gaps [68]. In the absence of an industry-wide standardized evaluation framework, the practical deployment of generative AI remains an issue that needs to be addressed.

## 3. Specific Applications of Four Typers of AI Technologies in the Aquatic Priducts Industry

**Figure 6:**
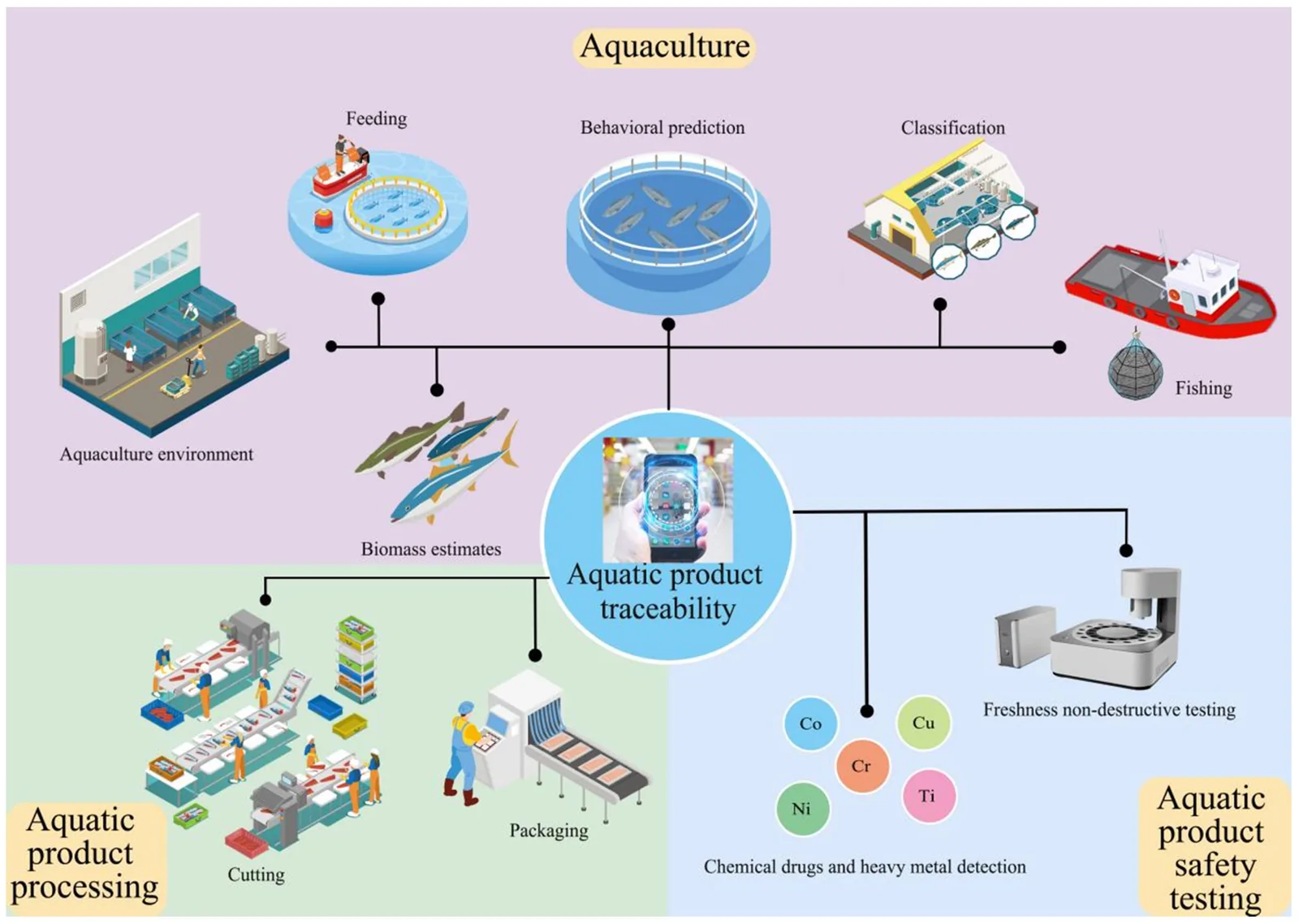
The practical application of AI technology in aquaculture, processing, safety testing, and traceability.

The application of AI technology in the aquatic products industry is mainly reflected in four major industries: aquaculture, aquatic product processing, safety detection of aquatic products, and traceability of aquatic products (Figure 6) and (Table 2). In aquaculture operations, key activities include monitoring the farming environment, fishing, estimating biomass, predicting fish behavior, and feeding. After aquaculture, aquatic products move to the processing stage, where they undergo cutting and then packaging. Subsequently, safety detection is carried out, involving nondestructive testing for freshness. The final traceability segment demonstrates the origin-tracing mechanism, where the mobile phone symbolizes the medium for accessing traceability data.

### 3.1. Application of AI Technology in Aquaculture

#### 3.1.1. Application of AI Technology in Aquaculture Environment

In aquaculture, aquatic organisms necessitate stringent water quality and environmental conditions. Variations in environmental parameters, such as dissolved oxygen levels and pH values, have a direct impact on the normal growth and development of fish [2]. Traditional aquaculture environments rely on manual data collection and detection as well as fixed water quality sensors, which is not only inefficient but also does not cover the entire water area. However, AI-based water quality management can help maintain optimal aquaculture conditions through real-time monitoring and active adjustment [3]. To predict dissolved oxygen (DO) and water temperature (WT) parameters in aquaculture environments, Zhou et al. [63] developed a deep learning-based predictive model incorporating a PID error correction mechanism. This corrector calculates prediction errors by comparing feedback with actual measurements, then adjusts current predictions to minimize deviations. However, the method still has limitations in long-term prediction accuracy, with overall errors remaining relatively high. Reference [14] proposed an improved fuzzy PID control system based on a residual-setting gray model to achieve advanced oxygen concentration control. Compared with traditional PID and fuzzy PID algorithms, the proposed control system is more effective in handling nonlinear, time-varying, and time-delay problems. This shows that the integration of predictive AI and control AI demonstrates significant potential in the field of aquaculture environment.

#### 3.1.2. Application of AI Technology in Aquatic Product Feeding

Feed plays a vital role in aquaculture, serving as a key factor in ensuring the healthy growth of fish, shrimp, and other aquatic organisms. High-quality feed not only provides essential nutrients but also enhances immunity and accelerates growth rates, thereby improving farming efficiency and economic returns. Therefore, selecting appropriate feeding plans and methods is crucial for successful aquaculture. Traditional manual feeding methods, lacking effective techniques, often result in high labor intensity while causing issues like feed waste and water quality deterioration [2]. The integration of advanced artificial intelligence techniques with sensor networks and data analytics significantly enhances the efficacy of feed delivery systems. The fundamental technology underpinning this system involves the amalgamation of underwater imaging and sonar technology to monitor fish behavior and feeding dynamics [25,108]. Wang et al. [113] employed computer vision and sonar technologies to gather and analyze data, enabling the timely adjustment of feeding plans. This responsive feeding strategy effectively mitigates the risks of overfeeding and underfeeding, optimizes growth rates, reduces operational costs, and minimizes nutrient runoff into aquatic environments. Ref. [108] proposed a feeding control method based on near-infrared computer vision and a neural fuzzy model. Compared with traditional feeding methods, this approach showed no significant difference in promoting fish growth, but it could reduce feed conversion ratio (FCR) by 10.77% and also decrease water pollution. Compared with traditional feeding methods, the introduction of perceptual AI technology significantly enhances the accuracy and rationality of feeding protocols.

#### 3.1.3. Application of AI Technology in Aquatic Product Behavioral Prediction

In the farming process, fish exhibit complex behaviors and can quickly adapt to perform related goals, such as feeding and interacting with their peers [114,115]. By analyzing fish behavior, aquaculture can help ensure optimal use of resources, control water quality, and assess fish health [113]. Typical fish behaviors include feeding behavior, aggressive behavior, disease status, and swimming behavior [116]. Zhao et al. [117], Cai et al. [93], and Ahmed et al. [118] have all developed the Trustworthy Multi-View Fish (TMVF) behavior recognition AI model to tackle core challenges in aquaculture, including irregular fish movement patterns and water surface reflections. The model shares a consistent core logic: using adaptive fusion as its foundation, it treats each viewpoint as a non-independent source domain. By mining multi-view correlation features, learning deep data relationships, and combining cross-fusion with Dirichlet distribution techniques, it resolves potential independence and conflict issues between viewpoints, ultimately generating predictions. This indicates that the accuracy of perceptual AI is a prerequisite for the reliability of predictive AI in predicting the behavior of aquatic products.

#### 3.1.4. Application of AI Technology in Aquatic Product Classification

Classification plays a crucial role in estimating species populations. Traditional biological classification methods for aquatic organisms typically rely on visual characteristics such as shape, color, and size through manual identification. However, this approach requires substantial human and material resources. Moreover, the distinct underwater environment differs fundamentally from terrestrial ecosystems, making comprehensive studies challenging to conduct effectively in aquatic settings [119]. AI, particularly predictive AI technology like support vector machines and convolutional neural networks, has been employed to automate and enhance the precision of classification processes [120,121,122]. These artificial intelligence models are developed utilizing extensive datasets, encompassing images and biometric data, to differentiate between species by analyzing morphological characteristics, including body shape, fin configuration, and coloration [116,123]. Hu et al. [22] used the combination of computer vision technology and multiple support vector machines (MSVM) to classify six different fish species. The findings indicated that the most effective classification model for recognizing fish species comprised a feature extractor operating in the wavelet domain, utilizing the Bior4.4 wavelet filter within the HSV color space, coupled with a Directed Acyclic Graph Multi-class Support Vector Machine (DAGMSVM) classifier employing a one-to-one algorithm. The convolutional neural network (CNN)-based approach proposed by [120] features a unified and versatile training architecture, demonstrating excellent performance in identifying four species of carp.

#### 3.1.5. Application of AI Technology in Aquatic Product Fishing

Traditional fishing technology lacks accurate understanding and regulation of resources, such as overfishing, destruction of coral reefs, algal beds, and other habitats, accidental capture, and ineffective fishing [124]. Liu et al. [96] developed a reinforcement learning-based dynamic coordination system for krill distribution and trawling operations. By employing neural networks to predict operational parameters and optimize fishing routes, the system increased krill density within the net by 8.4% while advancing fishing automation. Kunimatsu et al. [97] developed a spatiotemporal machine learning framework that integrates environmental variables with fish distribution to predict yield per unit area. Although limited to Japanese fishing grounds, this approach effectively reduces biases in population abundance index. The role of AI in advancing fishing is to resolve the core contradictions of traditional models—enhancing resource utilization efficiency through data-driven decision-making while reducing ecological damage with intelligent equipment.

#### 3.1.6. Application of AI Technology in Aquatic Product Biomass Estimates

The estimation of fish biomass encompasses the processes of counting, weight assessment, and length measurement, all of which are essential from the initial introduction of fry through to the final sale of the fish. Fish biomass is one of the parameters for in-depth understanding of fish and environmental health [26]. The accurate estimation of biomass provides data support for accurate feeding and aquaculture management. The conventional approach involves manual handling, which can inflict varying levels of harm on the fish, thereby adversely affecting their growth and marketability [2]. Computer vision and sonar technologies, as non-contact measurement methods, are extensively utilized due to their advantages of rapid processing speed and high efficiency [116]. To effectively address challenges such as occlusion, body deformation, and rapid fish movement, Cook et al. [94] employed ARIS imaging sonar for length estimation of small fish, achieving a measurement error <10%. Comparative analysis with DIDSON imaging sonar revealed that ARIS demonstrates higher precision at high detection frequencies. However, the study also noted that imaging sonar-based estimates were significantly less accurate than those obtained through stereo camera technology. This finding suggests that imaging sonar can complement other sampling techniques, with combined applications enhancing overall accuracy. Zhang et al. [98] developed a rapid, accurate, and fully automated fish biomass estimation system by integrating deep learning and computer vision technologies. The system measures fish body length and height using visual technology, then calculates weight through specific algorithms. Experimental results demonstrated that the DL-YOLO algorithm can efficiently detect and extract high-quality images of moving fish schools in real-time, effectively overcoming challenges like body occlusion, curvature, and non-orthogonal angles. Compared to YOLOv5n and YOLOv6n, the system achieved a 9.33% improvement in mAP@0.5:0.95 metrics. In an article by Tseng et al. [99], an automated measurement method was developed for determining the length of a fish’s nose-to-tail fork in complex images. The approach begins with acquiring images of fish bodies and color patches with known dimensions. A CNN classifier was then developed and applied to detect the fish’s head, tail fork, and color patch regions. Subsequently, image processing techniques were used to identify the nose and fork points in the head and tail fork areas, respectively. The fish’s length was estimated as the distance between these two points using the length-to-pixel ratio. This demonstrates that integrating perceptual AI with other AI technologies holds significant potential for aquatic biomass estimation.

Overall, AI research in the aquaculture sector exhibits a clear trend toward thematic concentration, primarily focusing on biomass estimation and behavior recognition. In contrast, research in other equally important yet more challenging areas—such as seedling quality assessment, clinical sensitivity for early disease detection, and breeding management—remains significantly underdeveloped [125]. This situation may contribute to an imbalance across industries during the advancement of AI applications in aquaculture. Furthermore, comparability across different studies is extremely low: studies employ diverse livestock species, stocking densities, water environments, and image acquisition conditions, and there is a shortage of a unified benchmark dataset [3].Therefore, in the aquaculture sector, it is crucial to establish a public benchmark dataset and promote the open sharing of code and data.

### 3.2. Application of AI Technology in Aquatic Product Processing

AI has substantially enhanced the efficiency, precision, and sustainability of aquaculture operations within the domain of aquatic product farming. Additionally, it has streamlined the processing stages, ranging from cutting to packaging, in the realm of aquatic product processing. This technological advancement has led to a decreased demand for labor, minimized post-harvest losses of aquatic products, and ensured the maintenance of high standards of product quality [101,126]. has the potential to facilitate real-time analysis of the quality of aquatic products by assessing parameters such as size, weight, and physical condition. Additionally, AI can enhance the precision of cutting and expedite the packaging process of these products [101,127]. These methods enhance the uniformity of product quality and diminish dependence on manual inspection, thereby mitigating the potential for human error.

#### 3.2.1. Application of AI Technology in Aquatic Product Cutting

Intelligent cutting is based on traditional cutting and advanced technology to realize the automation and intelligence of aquatic processing. In aquatic processing, the main cutting methods are metal blade cutting, water jet cutting (WJC), and ultrasonic-assisted cutting (UVAC) [127]. Metal blade cutting poses risks of contaminating aquatic products and compromising their integrity. To overcome these challenges, advanced technologies such as water jet cutting and ultrasonic-assisted cutting have been developed. These techniques facilitate ultra-precise cutting while maintaining the structural integrity and aesthetic quality of the products. Water jet cutting employs high-pressure or abrasive water jets to process aquatic materials. When combined with robotic arms, this method is noted for its rapid execution and exceptional precision in managing delicate products [128]. Ultrasonic cutting is recognized for its capability to achieve precise incisions without causing compression or damage to the surrounding tissue [126]. Importantly, both water jet and ultrasonic-assisted cutting do not produce heat, which helps maintain the quality and texture of aquatic products. To further reduce reliance on manual labor, the emergence of intelligent technologies such as computer vision and robotics has provided cutting-edge slicing solutions for aquatic product processing. Jang & Seo (2023) [100] proposed a computer vision-based approach that constructs 3D fish models through image processing and random sampling consistency techniques, extracts volumetric features, and establishes a neural network using machine learning and weight data to predict cutting points based on target weight. Azarmdel et al. (2021) [129] developed a computer vision-driven cutting robot that measures trout dimensions and extracts precise cutting points, both leveraging AI technology to enable accurate cutting in aquatic processing. This demonstrates that the integration of perceptual and control AI enables faster, more objective, and more efficient execution of aquatic product cutting.

#### 3.2.2. Application of AI Technology in Aquatic Product Packaging

Packaging plays a vital role in aquatic product processing, because it not only affects the storage and protection of aquatic products, but also affects the quality and shelf life of aquatic products [130]. At present, the packaging process in the aquatic product processing production line mainly relies on manual operation. Although this technology can protect food from pathogens, oxygen damage, and mechanical damage, manual packaging is limited by low automation, which seriously hinders the efficiency of aquatic product packaging [131]. In recent years, the integration of AI technology with packaging processes has introduced innovative concepts for the packaging of aquatic products. The deployment of robotic arms within sterile, pollution-free, unmanned production facilities enhances the hygiene and safety of food production processes. This integration mitigates the risk of cross-contamination, augments production efficiency, and ensures the quality and reliability of the final product [132]. The integration of machine vision technology into robotic arms enhances their autonomy and intelligence, thereby optimizing production line efficiency and quality while minimizing human error and waste [133]. Zhang et al. [101] developed an automated precision solution for oyster harvesting and packaging by integrating visual tracking algorithms with ROS robotic arm control technology, achieving dual guarantees of efficiency and quality. The system employs a deep learning-based YOLOv5-SORT intelligent perception model to perform real-time oyster detection, tracking, counting, and grasping-position prediction. During static harvesting, the robotic arm achieved an average success rate of 94.44% with a single-operation duration of 7.8 s. System evaluations demonstrated significant optimizations in layout design, operational efficiency, and automated packaging performance. The integration of perceptual AI technology with robotic arms significantly enhances the efficiency, safety, and stability of aquatic product packaging.

### 3.3. Application of AI Technology in Aquatic Product Safety Testing

During the transportation and storage of aquatic products, their quality gradually deteriorates over time until they become unfit for consumption. Therefore, identifying effective safety indicators for aquatic products is crucial. Aquatic product safety is influenced by multiple factors, including species, temperature, microorganisms, feed, season, reproductive cycle, harvesting methods, cutting techniques, and geographical location. Freshness, microbial levels, and toxic elements are key indicators for assessing aquatic product safety. Therefore, this section will demonstrate the application of artificial intelligence technology in aquatic product safety testing through freshness non-destructive testing and chemical/heavy metal analysis.

#### 3.3.1.  Application of AI Technology in Aquatic Product Non-Destructive Testing

Freshness non-destructive testing (NDT) has become a crucial factor in aquatic product quality assessment, both domestically and globally. Manual inspections remain vulnerable to human factors such as errors, subjectivity, and fatigue, driving the growing demand for non-destructive, simple, rapid, and cost-effective NDT methods. With advancements in computer science, spectroscopy, sensor technology, and related industries, researchers have developed three primary NDT technologies through visual analysis (Table 3): spectral techniques (including near-infrared spectroscopy, hyperspectral imaging, Raman spectroscopy, and fluorescence spectroscopy), sensory technologies (such as electronic nose, electronic tongue, and computer vision), and sensor technologies (comprising colorimetric sensors and gas detectors) [134]. This demonstrates that current aquatic product freshness detection technologies are built around two core architectures: perceptual AI for feature acquisition and predictive AI for modeling analysis. Perceptual AI leverages multimodal technologies including spectroscopy, sensor data, and computer vision to capture physicochemical and visual information, providing foundational data support for detection. Predictive AI establishes quantitative analysis frameworks through traditional machine learning and deep learning models, enabling precise measurement of key freshness indicators such as TVB-N and TVC. While model performance varies across different technical approaches, deep learning models consistently demonstrate superior detection accuracy and efficiency. This highlights the pivotal role of technological convergence in advancing aquatic product freshness detection capabilities.

#### 3.3.2. Application of AI Technology in Aquatic Product Chemical Drugs and Heavy Metal Detection

The bioaccumulation of chemical substances, including pesticides, veterinary drugs, and heavy metals, represents a significant environmental challenge. These compounds are highly toxic to aquatic organisms and pose potential threats to human health via the food chain. Of particular concern are the environmental contamination issues arising from the illegal addition of prohibited antibiotics in aquaculture, such as metabolites of furazolidone, chloramphenicol, malachite green (MG), and sodium nitrophenolate. Consequently, the development of highly sensitive, precise, and rapid detection methods utilizing AI technology has become imperative. This approach not only ensures the safety of aquatic products but also effectively addresses the limitations inherent in traditional chemical testing methods. At present, AI-based methodologies have significantly advanced the assessment of pollutant distribution, bioaccumulation factors, persistence, bioaccumulation, and toxicity in fish and seafood samples. [95,140]. Jiao et al. [134] developed a highly sensitive fluorescence sensor utilizing dual-emission molecularly imprinted polymers, enhanced by radio frequency algorithms, for the rapid detection of perchloromethane in fish samples. Through the analysis of RGB color values from fluorescence images using the random forest algorithm, they achieved precise concentration measurements, evidenced by an R^2^ value of 0.9958, employing Software Python (3.7.4, anaconda, on win32). Liu et al. [104] developed a wide-color-range fluorescent visual test paper based on quantum dot-molecular imprinting sensors and graphene quantum dots, which can detect chlorantraniliprole in fish samples with a detection limit of 5.0 μg/kg. Subsequently, a support vector regression (SVR) model was developed utilizing the RGB values of fluorescence colors alongside corresponding concentration data, resulting in a highly accurate prediction of chlorantraniliprole content, evidenced by a correlation coefficient of 0.98. This advancement underscores the substantial enhancement in detection and analysis accuracy afforded by machine learning models. Furthermore, the presence of heavy metals in aquatic products remains a significant public health concern, attributed to their non-biodegradable nature.

However, in the context of chemical drug and heavy metal detection, machine learning models are typically trained on limited sample datasets, resulting in broad prediction confidence intervals that often fail to meet the stringent accuracy requirements imposed by food safety regulations. More importantly, AI-based food safety detection systems face the dilemma that high accuracy does not necessarily equate to high practicality. As noted in the review by Bittsanszky et al. [141], the greatest potential for AI in food safety management lies not in broad, autonomous systems, but in targeted tools—such as camera-based preliminary screening, sensors for cold chain monitoring, digital traceability systems, and the documentation of alerts and corrective actions within HACCP frameworks.

### 3.4. Application of AI Technology in Aquatic Product Traceability

With the global expansion of the aquatic products industry and growing consumer concerns about food safety, traceability in aquatic product supply chains has become crucial for ensuring quality and sustainable industry development. Traditional manual documentation and fragmented management models can no longer meet the demands of full-chain transparency. The deep integration of Artificial Intelligence (AI), Internet of Things (IoT), blockchain, and other technologies has revolutionized traceability solutions in the aquaculture industry [142]. AI technology is becoming increasingly valuable for augmenting traceability systems through the integration of data across all stages of breeding, processing, and transportation.

Blockchain technology ensures immutability of records once committed to the ledger, providing tamper-evident audit trails that enhance trust among supply chain participants [143]. However, it is essential to recognize that blockchain does not inherently guarantee the correctness or truthfulness of data at the point of entry [144]. If inaccurate, incomplete, or fraudulent information is recorded on-chain, the blockchain will preserve that erroneous record with the same integrity as a correct one. Therefore, the reliability of a blockchain-based traceability system depends critically on the data quality and verification mechanisms employed at the data capture stage, rather than on the blockchain layer alone.

The IoT constitutes a comprehensive framework encompassing IoT devices, communication technologies, the Internet, and data storage, processing, and monitoring systems. Collectively, these components facilitate the generation of insights and the identification of trends across diverse industries [145]. Gao et al. developed an Internet of Things-based fish farming and tracking system that enables automated water quality management and supports the tracking of freshwater fish farming and sales [9]. Li et al. [127] concluded that a digital monitoring system utilizing the IoT is capable of real-time surveillance of production status. Furthermore, the implementation of real-time monitoring via an IoT platform enhances traceability and food safety in the context of fish processing.

## 4. Future Development

With the ongoing advancements in the aquaculture, processing, and transportation industries, there is an increasing demand for enhanced food safety and quality monitoring standards. Future research should prioritize the following areas:

### 4.1. The Rapid Development of Multimodal Perceptual AI Technology

Current single-modal technologies still have limitations in complex underwater environments (such as highly turbid waters and biologically dense areas). Future efforts should focus on developing more efficient multimodal fusion algorithms, with key solutions to issues such as cross-modal data alignment, dynamic weight allocation, and real-time processing. For example, integrating the self-attention mechanism of Transformers with Graph Neural Networks (GNNs) can achieve in-depth correlation of visual, acoustic, and chemical sensing data, thereby improving the reliability of perception in extreme environments.

### 4.2. Optimization of Long-Term Performance and Interpretability of Predictive Models

Current models exhibit instability in long-term forecasting, particularly concerning quarterly growth trends, and demonstrate vulnerability under complex disturbances, such as abrupt pollution events and climate change. In the future, it is necessary to combine causal inference with deep learning to construct models with both long-term predictive capability and interpretability. For instance, by introducing hybrid models with physical constraints (such as physics-informed neural networks), the scientific credibility of predictions can be enhanced. Furthermore, the application of few-shot learning and transfer learning will reduce the dependence of models on large-scale labeled data, facilitating rapid deployment in different scenarios.

### 4.3. Autonomy and Adaptive Upgrading of Control AI

Existing control strategies have limited adaptability in multi-objective optimization (such as balancing yield and energy consumption) and under dynamic interferences (such as equipment failures and sudden environmental changes). Future endeavors should focus on developing autonomous control systems based on Reinforcement Learning (RL), which can realize dynamic optimization of strategies through real-time interaction with the environment. Meanwhile, combining digital twin technology to construct virtual scenarios will enable offline simulation and online iteration of control strategies, improving the regulation accuracy and robustness in complex scenarios.

### 4.4. Generative AI with the Industrial Chain

The application of generative AI in the aquatic products industry is still in its infancy. In the future, its potential can be explored in the following directions: generating virtual aquaculture scenarios based on multi-source data for technical verification and personnel training; designing new feed formulations and aquaculture models through generative models to accelerate the innovation process; and combining blockchain with generative AI to realize Intelligent artificial generation and verification of traceability data, thereby enhancing supply chain transparency.

## 5. The Limitations of Four Categories of Artificial Intelligence Technologies in the Aquatic Products Industry

The application of artificial intelligence in the aquaculture sector faces certain challenges, which are often the result of a lack of targeted research. At the data level, biological fouling, corrosion, turbidity fluctuations, and sensor drift in aquatic environments not only generate significant noise and labeling errors but also induce time-dependent distribution shifts, causing existing models to become rapidly ineffective. Additionally, variations among farms—such as differences in water quality, feeding practices, stocking density, lighting conditions, and species—lead to significant cross-domain generalization failures; however, existing transfer learning approaches have not yet been validated at a commercial scale. At the application level, the characteristics of mainstream deep learning models conflict with empirical intuition, and the absence of interpretable methods tailored to aquaculture scenarios means that systems with high accuracy are still being rejected for deployment.

More profound constraints stem from the security, governance, and institutional dimensions. IoT connectivity exposes farming systems to the risk of adversarial attacks—potentially enabling the tampering of quality inspection results or triggering false alarms—while sensitive production data lacks established encryption standards or robust privacy protection frameworks. From a regulatory standpoint, food safety authorities currently lack evaluation guidelines for AI-driven decision-making; legal accountability for erroneous judgments remains unresolved; and ambiguity of responsibilities among developers, deployers, and regulatory agencies may pose greater constraints than any technical bottleneck. These risks do not exist in isolation but collectively form a complex set of implementation barriers spanning from the perception layer to the institutional layer; there is an urgent need for interdisciplinary collaborative research and the joint development of industry-wide standards—otherwise, technological advancements will be difficult to translate into tangible industrial value.

## 6. Conclusions

This review has examined the current state and emerging trajectories of artificial intelligence across four functional domains in aquaculture: perception, prediction, control, and generation. While each domain demonstrates notable progress, their efficacy is not universal but highly contingent on data quality, species-specific biology, environmental variability, and the rigor of validation design. Perception AI shows promise in monitoring water quality and organism health, yet its reliability remains sensitive to optical conditions, sensor calibration, and annotation consistency. Predictive models can capture complex biological dynamics under controlled settings, but their generalizability is frequently compromised by distributional shifts across farms, seasons, and genetic lines. Control systems exhibit adaptive potential in simulation or pilot studies, but face significant challenges when deployed in unstructured, stochastic environments where safety and interpretability are paramount. Generative AI, though nascent, offers exploratory value for synthetic data augmentation and scenario modeling, yet lacks empirical grounding in real-world aquaculture workflows.

Critically, the gap between laboratory-reported performance and field-deployable robustness remains substantial. As detailed in Section 5, practical implementation is hindered not merely by algorithmic limitations but by systemic barriers: pervasive data heterogeneity and labeling noise; domain shift due to site- and species-specific contexts; the opacity of black-box models that undermines stakeholder trust; escalating computational and energy costs at the edge; unresolved questions regarding liability and regulatory compliance; and cybersecurity vulnerabilities in increasingly connected infrastructure. These constraints are not peripheral—they define the operational reality of modern aquaculture and must be central to any assessment of AI’s applicability.

Moving forward, advancement should prioritize constraint-aware development over incremental accuracy gains. Key priorities include establishing standardized, cross-domain validation protocols that explicitly test for environmental and biological variability; designing models that are robust to data scarcity and distributional drift rather than optimized solely for benchmark performance; integrating explainability and uncertainty quantification as core requirements, not afterthoughts; co-developing solutions with industry stakeholders to align technical capabilities with practical needs such as cost, maintainability, and interoperability; and fostering interdisciplinary collaboration among biologists, engineers, data scientists, and policymakers to address socio-technical dimensions of deployment.

Ultimately, AI’s transformative potential in the aquatic products industry is neither guaranteed nor uniformly attainable. Its value will be realized only through sustained attention to the contextual, operational, and ethical constraints that shape real-world adoption. Future success should therefore be measured not by peak performance in idealized settings, but by the degree to which AI systems demonstrate adaptability, reliability, and trustworthiness within the complex, variable, and often resource-limited conditions of actual aquatic production.

## Figures and Tables

**Table 2:** The application of AI technology in the aquatic products industry.

Types of AI	Applications	AI Technology	Purpose	Model Types	Model Evaluation	References
Perceptual AI	Biomass estimates	Computer vision	Monitor mariculture from remote sensing images	YOLOv5	Achieves 91% precision	[12]
	Biomass estimates	Computer vision	Propose a deformable 2D *Tuna* model to realize visual recognition	FEI	90.6% accuracy	[23]
	Behavior prediction	Computer vision	Monitor local unusual behaviors of fish schools in intensive aquaculture	RNN	98.91% accuracy	[24]
	Behavior prediction	Sonar signal	Realize real-time monitoring of fish in marine aquaculture	LoRa Add-on Module	QoS over 90%	[29]
	Behavior prediction	Sonar signal	Establish the *Turbot*’s link between sound signals and feeding activity	Linear regression model	R^2^ = 0.832	[30]
	Behavior prediction	Computer vision	Optimize fish feeding behavior recognition and solve issues of data redundancy, complex models, and low efficiency	YOLOv8, ResNet	83.33% accuracy	[93]
	Biomass estimates	Sonar signal	Evaluate ARIS application value in turbid and low-light environments	ARIS, linear mixed model	0.3–9.6% measurement error	[94]
	Chemical drugs and heavy metal detection	Sensor	To achieve rapid, sensitive qualitative and quantitative analysis of toxic substances in aquatic products	PCA, KNN	R^2^ = 0.9944	[95]
	Behavior prediction	Multimodal data fusion	Address the issues that traditional single-modal assessment of fish feeding intensity is vulnerable to environmental interference.	MMFINet (Pre-training S3D mode, PANNS-MobileNetV2, TCN)	97.6% accuracy	[43]
Predictive AI	Classification	ML	Fish classification	DAG-VBMSVM	97.77% accuracy	[22]
	Fishing	DL	Improving the efficiency of fishing for *Antarctic krill* and promoting the automation of trawling operations	Q-learning, GAM	GAM explains 74.7% of the deviation	[96]
	Biomass estimates	ML	Optimize the accuracy of fishery resource abundance estimation	RF, GBDT, TabNet	R^2^ = 0.668	[97]
	Biomass estimates	DL	Develop a fully automatic and accurate estimation system.	DL-YOLO, CatBoost	R^2^ = 0.98, MRE = 2.87%	[98]
	Biomass estimates	DL	Address the time-consuming and subjective issues of manual measurement	CNN	98.78% accuracy	[99]
	Cutting of aquatic products	DL	Address the dangers and time-consuming nature of manual fish cutting in fish processing and the large errors of automated equipment	Artificial neural networks	MRE is about 3%	[100]
	Aquatic product packaging	DL	Address the low efficiency and high contamination risk of manual raw oyster packaging.	YOLOv5	Success rate of grasping is 94.44%	[101]
	Freshness testing	ML	Build models to simultaneously predict indicators like TBA and TVB-N under 0 °C storage.	MLR, SVM	MLR model R^2^ = 0.9989−0.9997, SVM model R^2^ = 0.8423–0.9888	[102]
	Freshness testing	DL	Address the cumbersome and time-consuming issues of *Tilapia* cold storage freshness detection	CNN	92.31% accuracy	[103]
	Chemical drugs and heavy metal detection	ML	Achieve rapid and sensitive detection of propazine in fish and seawater samples.	SVM	R^2^ = 0.98	[104]
	IoT-based intelligent control and traceability system	ML	Realize water quality prediction, intelligent equipment regulation, and full-process traceability of aquatic products.	Cubist, RF, GBM, M5 model tree	R^2^ = 0.929	[105]
	Freshness testing	Multimodal data fusion	Address the issue of difficult non-destructive monitoring of *Sturgeon*’s respiratory stress state during waterless low-temperature transportation.	LR, BPNN, SVR, RBFNN	R^2^ = 0.9544	[44]
	Classification	Multimodal data fusion	Address the issues of low accuracy and vulnerability to environmental interference in traditional single-modal fish stress classification	RTI-Net	94.43% accuracy	[106]
	Behavior prediction	Multimodal data fusion	Address the issue of low accuracy in single-modal fish behavior recognition caused by dim lighting and environmental noise in aquaculture environments.	SEResNet50 backbone + MIF module + TAP layer + U-shaped bilinear fusion structure	94.25% accuracy	[107]
Control AI	Aquaculture environment	Fuzzy logic control system	Improve oxygen control stability and anti-interference.	GM-IPSO-GRU prediction model and IGWO-optimized fuzzy PID control model	R^2^ = 0.981–0.978	[14]
	Feed feeding	Fuzzy logic control system	Realize automatic feeding decisions based on fish appetite.	ANFIS decision model	98% accuracy	[108]
	Aquaculture environment	Fuzzy logic control system	Achieve precise oxygen regulation and ensure fish survival and quality.	Optimized PID control model	MAE = 0.0409, R^2^ = 0.981	[14]
	Feed feeding	Intelligent system control	Realize intelligent decision-making for feeding strategies.	YOLOv5, FNN	Recall reaching 80–90%	[109]
	Aquaculture environment	Model predictive control	Address the water quality deterioration caused by high water circulation rate in recirculating aquaculture systems.	NMPC	/	[85]
	Aquaculture environment	Intelligent system control	Address the issues of significant overshoot and weak adaptability in traditional PID controllers, which are induced by nonlinear factors.	DE-RBF-PID control model	Adjustment time was only 650 s	[110]
Generative AI	Biomass estimates	GAN	Address the difficulty in training deep learning models caused by the small scale.	AE-GAN	83.8% accuracy	[89]
	Behavior prediction	LLM	Address the issues of scarce textual data and uneven quality in the field of aquaculture disease prevention and control.	TDA-GLM	94.86% accuracy	[111]
	Cutting of aquatic products	VAE, GAN	Address the issues of time-consuming and subjective manual segmentation of Atlantic salmon ovary ultrasound.	U-Net + VAE/GAN	The individual average Dice coefficient was 0.885.	[90]
	Aquaculture environment	ML, LLM	Address the issues of traditional aquaculture relying on expert experience, low automation, large decision errors.	RAG-LLM, YOLOv8	RAG-LLM + DQN system has the best comprehensive performance, with decision error < 2%	[112]
	Behavior prediction	Diffusion model,	Achieve accurate recognition of 12 types of fish behavioral events, providing an efficient automated tool for marine ecological research and fishery management.	K-diffusion variant	Macro F1 68.1%	[91]

**ML** : Machine Learning; **DL** : Deep Learning; **LLM** : Large Language Model; **RAG-LLM** : Retrieval-Augmented Generation LLM; **YOLOv5/YOLOv8** : You Only Look Once (Version 5/8); **RNN** : Recurrent Neural Network; **PCA** : Principal Component Analysis; **KNN** : k-Nearest Neighbors; **MMFINet** : Multimodal Fusion Network; **S3D** : Separable 3D Convolutional Network; **PANNS** : Pre-trained Audio Neural Networks; **TCN** : Temporal Convolutional Network; SVM: Support Vector Machine; DAG: Directed Acyclic Graph; VB: Variational Bayes; **Q-learning** : A classic reinforcement learning algorithm in which an agent learns the optimal policy through interactions with its environment; **GAM** : Generalized Additive Model; **RF** : Random Forest; **GBDT** : Gradient Boosting Decision Tree; **DL-YOLO** : Deep Learning YOLO; **CatBoost** : Categorical Boosting; **CNN** : Convolutional Neural Network; **ANN** : Artificial Neural Network; **MLR** : Multiple Linear Regression; **SVM** : Support Vector Machine; **BPNN** : Back Propagation Neural Network; **SVR** : Support Vector Regression; **RBFNN** : Radial Basis Function Neural Network; **RTI-Net** : A network model designed for multimodal data fusion classification; **SEResNet50** : Squeeze-and-Excitation ResNet 50; **MIF** : Multi-scale Information Fusion; **TAP** : Task-Aware Pooling; **GAN** : Generative Adversarial Network; **AE-GAN** : Autoencoder-GAN; **GLM** : A Graph Language Model; TDA: Topological Data Analysis; **VAE** : Variational Autoencoder; **U-Net** : A convolutional neural network with a U-shaped symmetric structure; **K-diffusion** : A variant of generative algorithms based on diffusion models; **LoRa** : Long Range; **ARIS** : Acoustic Resonance Imaging Sonar; **GM:** Grey Model; IPSO: Improved Particle Swarm Optimization; GRU: Gated Recurrent Unit; **IGWO** : Improved Grey Wolf Optimizer; **PID** : Proportional-Integral-Derivative; **ANFIS** : Adaptive Neuro-Fuzzy Inference System; **FNN** : Feedforward Neural Network; **NMPC** : Nonlinear Model Predictive Control; DE: Differential Evolution; RBF: Radial Basis Function; **DQN** : Deep Q-Network; **QoS** : Quality of Service; **MRE** : Mean Relative Error; **MAE** : Mean Absolute Error; **TBA** : Thiobarbituric Acid; **TVB-N** : Total Volatile Basic Nitrogen.

**Table 3:** Application of AI technology in aquatic product freshness non-destructive testing.

Algorithm	Purpose	Sample	Model/Algorithm	Key Indicator	Performance	References
Hyperspectral imaging	Hyperspectral imaging combined with a LDNN and least squares support vector machine was used to predict the TVB-N of *Rainbow* trout fillets stored at 4 ± 2 °C for 12 days, respectively.	*Rainbow* trout fillets	Linear Deep Neural Network, PLSR, LS-SVM	TVB-N	The RP^2^, RSMEP, and RDP of LDNN are 0.853, 3.159, and 3.001, respectively, while the RMSEP and RP^2^ of the least squares support vector machine are 2.63 and 0.897, respectively.	[135]
NIRS	One-dimensional convolutional neural network, partial least squares regression, and various preprocessing methods were compared with near-infrared spectroscopy to determine the optimal model for detecting the freshness of crayfish.	Crayfish	1D-CNN, PLSR	TVB-N, TVC	The one-dimensional convolutional neural network achieved the best model performance in terms of TVB-N and TVC, with Rp^2^ values of 0.9397 and 0.9318, and RPD values of 2.8279 and 2.7560, respectively.	[78]
Raman spectroscopy	A convolutional neural network is used to model Raman spectral data for the rapid classification of the freshness grades of perch fillets.	Sea bass fillets	CNN, PLS-DA, SVM	TVB-N, Protein degradation	The average accuracy is increased to 90.6%, and the testing time is reduced by 0.44.	[136]
Fluorescence spectroscopy	Fluorescence spectroscopy combined with a long short-term memory network model to evaluate the freshness of bighead carp heads	Bighead carp heads	LSTM	TVB-N, TBARS, K-value, TVC	The R^2^ value exceeds 0.884, and the relative error compared with the direct-sale cold chain in supermarkets is less than 10%.	[137]
Electronic nose	To realize aquatic product freshness classification and TVC prediction using electronic nose combined with machine learning, and verify the effectiveness of a low-sensor system	Double-whip threadfin bream, octopus, cuttlefish, squid	Random Forest, Decision Tree, KNN, AdaBoost, Gradient Tree Boosting, and Support Vector Machine	TVC	The R^2^ and RMSE are 0.995 and 0.003, respectively.	[138]
Computer vision technology	Prediction of the freshness of salmon stored at 0 °C using multiple linear regression and support vector regression models based on computer vision detection.	Salmon	MLR, SVR	TVB-N, TBARS, TVC, K-value	The multiple linear regression model has high correlation coefficients (R^2^ = 0.9989–0.9997 and F-values = 186.26–589.42) for TBA, TVB-N, TVC, and K-value.	[102]
Colorimetric sensor	Detection of the indole-producing ability of pathogenic and spoilage bacteria in shrimp species using deep learning combined with a fish colorimetric sensor.	Shrimp	DCNN	TVB-N, TVC	The accuracy of the VGG16 algorithm reaches a maximum of 97.89%.	[139]
Gas sensor	By integrating electronic nose and electronic tongue technologies with various machine learning algorithms, the freshness of mackerel can be predicted.	Tilapia	Convolutional Neural Network, PCA, BP network	TVB-N	Each algorithm has its own advantages. Among them, ANN, RFR, and XGBoost have better prediction performance for physicochemical indices, with RP^2^ being 0.929, 0.936, and 0.888, respectively.	[103]

Linear Deep Neural Network (LDNN); Total Volatile Basic Nitrogen (TVB-N); Partial Least Squares Regression (PLSR); Least Squares Support Vector Machine (LS-SVM); Near-Infrared Spectroscopy (NIRS); One-Dimensional Convolutional Neural Network (1D-CNN); Total Viable Count (TVC); Convolutional Neural Network (CNN); Partial Least Squares Discriminant Analysis (PLS-DA); Support Vector Machine (SVM); Long Short-Term Memory (LSTM); Thiobarbituric Acid Reactive Substances (TBARS); K-Nearest Neighbors (KNN); Multiple Linear Regression (MLR); Support Vector Regression (SVR); Thiobarbituric Acid Reactive Substances (TBARS); Deep Convolutional Neural Networks (DCNN); Principal Component Analysis (PCA); Back Propagation network (BP network); Artificial Neural Network (ANN); Random Forest Regressor (RFR); Extreme Gradient Boosting (XGBoost).

## Data Availability

The original contributions presented in this study are included in the article. Further inquiries can be directed to the corresponding author.

## References

[1] Fernando I.P.S., Jayawardena T.U., Wu J. (2023). Marine Proteins and Peptides: Production, Biological Activities, and Potential Applications. Food Innov. Adv..

[2] Wu Y., Duan Y., Wei Y., An D., Liu J. (2022). Application of Intelligent and Unmanned Equipment in Aquaculture: A Review. Comput. Electron. Agric..

[3] Yang H., Feng Q., Xia S., Wu Z., Zhang Y. (2025). AI-Driven Aquaculture: A Review of Technological Innovations and Their Sustainable Impacts. Artif. Intell. Agric..

[4] Yue K., Shen Y. (2022). An Overview of Disruptive Technologies for Aquaculture. Aquac. Fish..

[5] Rashvand M., Ren Y., Sun D.-W., Senge J., Krupitzer C., Fadiji T., Miró M.S., Shenfield A., Watson N.J., Zhang H. (2025). Artificial Intelligence for Prediction of Shelf-Life of Various Food Products: Recent Advances and Ongoing Challenges. Trends Food Sci. Technol..

[6] Russo G.L., Langellotti A.L., Torrieri E., Masi P. (2024). Emerging Technologies in Seafood Processing: An Overview of Innovations Reshaping the Aquatic Food Industry. Compr. Rev. Food Sci. Food Saf..

[7] Yu W., Ouyang Z., Zhang Y., Lu Y., Wei C., Tu Y., He B. (2025). Research Progress on the Artificial Intelligence Applications in Food Safety and Quality Management. Trends Food Sci. Technol..

[8] Cui F., Zheng S., Wang D., Tan X., Li Q., Li J., Li T. (2023). Recent Advances in Shelf Life Prediction Models for Monitoring Food Quality. Compr. Rev. Food Sci. Food Saf..

[9] Gao G., Xiao K., Chen M. (2019). An Intelligent IoT-Based Control and Traceability System to Forecast and Maintain Water Quality in Freshwater Fish Farms. Comput. Electron. Agric..

[10] Liu Z., Yu X., Liu N., Liu C., Jiang A., Chen L. (2025). Integrating AI with Detection Methods, IoT, and Blockchain to Achieve Food Authenticity and Traceability from Farm-to-Table. Trends Food Sci. Technol..

[11] Mustapha U.F., Alhassan A., Jiang D., Li G. (2021). Sustainable Aquaculture Development: A Review on the Roles of Cloud Computing, Internet of Things and Artificial Intelligence (CIA). Rev. Aquac..

[12] Quaade S., Vallebueno A., Alcabes O.D.N., Rodolfa K.T., Ho D.E. (2024). Remote Sensing and Computer Vision for Marine Aquaculture. Sci. Adv..

[13] Zeng J., Song Y., Cong P., Cao Y., Xu J., Xue C. (2024). A Systematic Review of Drying in Aquatic Products. Rev. Aquac..

[14] Zhang Y., Xiao X. (2024). Fuzzy PID Control System Optimization and Verification for Oxygen-Supplying Management in Live Fish Waterless Transportation. Inf. Process. Agric..

[15] Zhao S., Zhu M., Ding W., Zhao S., Gu J. (2020). Feed Requirement Determination of Grass Carp (*Ctenopharyngodon idella*) Using a Hybrid Method of Bioenergetics Factorial Model and Fuzzy Logic Control Technology under Outdoor Pond Culturing Systems. Aquaculture.

[16] Pallottino F., Violino S., Figorilli S., Pane C., Aguzzi J., Colle G., Nerio Nemmi E., Montaghi A., Chatzievangelou D., Antonucci F. (2025). Applications and Perspectives of Generative Artificial Intelligence in Agriculture. Comput. Electron. Agric..

[17] Du Y., Wang Z., Wei D. (2025). Advancing Tele-Perception: A Paradigm Shift from Traditional Non-Contact Sensing to Adaptive Embodied Artificial Intelligence Systems. Sci. Bull..

[18] Li W., Du Z., Xu X., Bai Z., Han J., Cui M., Li D. (2024). A Review of Aquaculture: From Single Modality Analysis to Multimodality Fusion. Comput. Electron. Agric..

[19] Wang G., Yu J., Xu W., Muhammad A., Li D. (2025). Automated Fish Counting System Based on Instance Segmentation in Aquaculture. Expert Syst. Appl..

[20] Zhou C., Xu D., Lin K., Sun C., Yang X. (2018). Intelligent Feeding Control Methods in Aquaculture with an Emphasis on Fish: A Review. Rev. Aquac..

[21] Zhang B., Huang W., Li J., Zhao C., Fan S., Wu J., Liu C. (2014). Principles, Developments and Applications of Computer Vision for External Quality Inspection of Fruits and Vegetables: A Review. Food Res. Int..

[22] Hu J., Li D., Duan Q., Han Y., Chen G., Si X. (2012). Fish Species Classification by Color, Texture and Multi-Class Support Vector Machine Using Computer Vision. Comput. Electron. Agric..

[23] Atienza-Vanacloig V., Andreu-García G., López-García F., Valiente-González J.M., Puig-Pons V. (2016). Vision-Based Discrimination of Tuna Individuals in Grow-out Cages through a Fish Bending Model. Comput. Electron. Agric..

[24] Zhao J., Bao W., Zhang F., Zhu S., Liu Y., Lu H., Shen M., Ye Z. (2018). Modified Motion Influence Map and Recurrent Neural Network-Based Monitoring of the Local Unusual Behaviors for Fish School in Intensive Aquaculture. Aquaculture.

[25] Li D., Du Z., Wang Q., Wang J., Du L. (2024). Recent Advances in Acoustic Technology for Aquaculture: A Review. Rev. Aquac..

[26] Li D., Hao Y., Duan Y. (2020). Nonintrusive Methods for Biomass Estimation in Aquaculture with Emphasis on Fish: A Review. Rev. Aquac..

[27] Testolin A., Kipnis D., Diamant R. (2022). Detecting Submerged Objects Using Active Acoustics and Deep Neural Networks: A Test Case for Pelagic Fish. IEEE Trans. Mob. Comput..

[28] Merchant N.D., Fristrup K.M., Johnson M.P., Tyack P.L., Witt M.J., Blondel P., Parks S.E. (2015). Measuring Acoustic Habitats. Methods Ecol. Evol..

[29] Hassan W., Føre M., Ulvund J.B., Alfredsen J.A. (2019). Internet of Fish: Integration of Acoustic Telemetry with LPWAN for Efficient Real-Time Monitoring of Fish in Marine Farms. Comput. Electron. Agric..

[30] Mallekh R., Lagardère J.P., Eneau J.P., Cloutour C. (2003). An Acoustic Detector of Turbot Feeding Activity. Aquaculture.

[31] Su X., Sutarlie L., Loh X.J. (2020). Sensors, Biosensors, and Analytical Technologies for Aquaculture Water Quality. Research.

[32] Tang X., Jiang H., Wen R., Xue D., Zeng W., Han Y., Wu L. (2024). Advancements and Challenges on SERS-Based Multimodal Biosensors for Biotoxin Detection. Trends Food Sci. Technol..

[33] Wei T., Shi Y., Sun J. (2025). A Multimodal Interaction-Based Approach to Situational Driving Anger Emotion Regulation. Expert Syst. Appl..

[34] Zhao Y., Qiu L., Yang Z., Chen Y., Zhang Y. (2025). MGF-GCN: Multimodal Interaction Mamba-Aided Graph Convolutional Fusion Network for Semantic Segmentation of Remote Sensing Images. Inf. Fusion.

[35] Ghamisi P., Gloaguen R., Atkinson P.M., Benediktsson J.A., Rasti B., Yokoya N., Wang Q., Hofle B., Bruzzone L., Bovolo F. (2019). Multisource and Multitemporal Data Fusion in Remote Sensing: A Comprehensive Review of the State of the Art. IEEE Geosci. Remote Sens. Mag..

[36] Li J., Hong D., Gao L., Yao J., Zheng K., Zhang B., Chanussot J. (2022). Deep Learning in Multimodal Remote Sensing Data Fusion: A Comprehensive Review. Int. J. Appl. Earth Obs. Geoinf..

[37] Rasti B., Ghamisi P. (2020). Remote Sensing Image Classification Using Subspace Sensor Fusion. Inf. Fusion.

[38] Pereira L.M., Salazar A., Vergara L. (2023). A Comparative Analysis of Early and Late Fusion for the Multimodal Two-Class Problem. IEEE Access.

[39] Boulahia S.Y., Amamra A., Madi M.R., Daikh S. (2021). Early, Intermediate and Late Fusion Strategies for Robust Deep Learning-Based Multimodal Action Recognition. Mach. Vis. Appl..

[40] Li G., Wang Y., Liu Z., Zhang X., Zeng D. (2023). RGB-T Semantic Segmentation with Location, Activation, and Sharpening. IEEE Trans. Circuits Syst. Video Technol..

[41] Jiao J., Wei Y., Jie Z., Shi H., Lau R., Huang T.S. (2019). Geometry-Aware Distillation for Indoor Semantic Segmentation. Proceedings of the 2019 IEEE/CVF Conference on Computer Vision and Pattern Recognition (CVPR), Long Beach, CA, USA, 15–20 June 2019.

[42] Torres Caceres V.A., Duffaut K., Yazidi A., Westad F., Johansen Y.B. (2024). Automated Well Log Depth Matching: Late Fusion Multimodal Deep Learning. Geophys. Prospect..

[43] Gu X., Zhao S., Duan Y., Meng Y., Li D., Zhao R. (2025). MMFINet: A Multimodal Fusion Network for Accurate Fish Feeding Intensity Assessment in Recirculating Aquaculture Systems. Comput. Electron. Agric..

[44] Zhang L., Li Y., Wang W., Feng H., Hu J., Zhang X. (2024). Non-Destructive Detection of Sturgeon Breath under Waterless Low Temperature Stress Using Microenvironment and Breath Angle Multi-Modal Sensing. Biosyst. Eng..

[45] Mohammadi E., Rahimian M., Panahi B. (2025). Bridging the Gap: Phage Manufacturing Processes from Laboratory to Agri-Food Industry. Virus Res..

[46] Raman R.K., Mohanty S.K., Bhatta K.S., Karna S.K., Sahoo A.K., Mohanty B.P., Das B.K. (2018). Time Series Forecasting Model for Fisheries in Chilika Lagoon (a Ramsar Site, 1981), Odisha, India: A Case Study. Wetl. Ecol. Manag..

[47] Çakır M., Oral O., Ural G.N., Yılmaz M. (2026). Mapping the Research Landscape of Machine Learning in Aquaculture: A Bibliometric Analysis with Forecasted Output by 2030. Aquaculture.

[48] You J., Hao G., Gan X., Chen R., Chen Y., Zhang Z., Sun A., Liu H., Shi X. (2024). Extreme Gradient Boosting-Enhanced Molecularly Imprinted Fluorescence Nanosensor for Rapid Identification and Visual Detection of Deltamethrin in Seawater and Aquatic Products. Sens. Actuators B Chem..

[49] Taparhudee W., Jongjaraunsuk R., Nimitkul S., Suwannasing P., Mathurossuwan W. (2024). Optimizing Convolutional Neural Networks, XGBoost, and Hybrid CNN-XGBoost for Precise Red Tilapia (Oreochromis Niloticus Linn.) Weight Estimation in River Cage Culture with Aerial Imagery. AgriEngineering.

[50] Wang Q., Lu H., Li F., Cheng Y.F. (2025). Advancing LightGBM with Data Augmentation for Predicting the Residual Strength of Corroded Pipelines. npj Mater. Degrad..

[51] Siddique M.A.B., Mahalder B., Haque M.M., Shohan M.H., Biswas J.C., Akhtar S., Ahammad A.K.S. (2024). Forecasting of Tilapia (Oreochromis Niloticus) Production in Bangladesh Using ARIMA Model. Heliyon.

[52] Dong X., Xu Z., Zhao H., Wu D., Qu B., Liu S., Xiao B. (2025). Predictive Modeling and Interpretability Analysis of Bioconcentration Factors for Organic Chemicals in Fish Using Machine Learning. Environ. Pollut..

[53] Baez-Villanueva O.M., Zambrano-Bigiarini M., Beck H.E., McNamara I., Ribbe L., Nauditt A., Birkel C., Verbist K., Giraldo-Osorio J.D., Xuan Thinh N. (2020). RF-MEP: A Novel Random Forest Method for Merging Gridded Precipitation Products and Ground-Based Measurements. Remote Sens. Environ..

[54] Saville R., Fujiwara A., Hatanaka K., Wada M., Yaman A., Puspasari R., Albasri H., Dwiyoga N. (2026). AI-Powered Decision Support System for Mariculture: Real-Time Fish Mortality Prediction with Random Forest. Aquac. Eng..

[55] Liu X., Wang Y., Chen T., Gu X., Zhang L., Li X., Tang R., He Y., Chen G., Zhang B. (2024). Monitoring Water Quality Parameters of Freshwater Aquaculture Ponds Using UAV-Based Multispectral Images. Ecol. Indic..

[56] Massaro A., Stella R., Negro A., Bragolusi M., Miano B., Arcangeli G., Biancotto G., Piro R., Tata A. (2021). New Strategies for the Differentiation of Fresh and Frozen/Thawed Fish: A Rapid and Accurate Non-Targeted Method by Ambient Mass Spectrometry and Data Fusion (Part a). Food Control.

[57] Duan Y., Li X., Zhang L., Chen D., Liu S., Ji H. (2020). Mapping National-Scale Aquaculture Ponds Based on the Google Earth Engine in the Chinese Coastal Zone. Aquaculture.

[58] Kanwal S., Abdullah M., Kumar S., Arshad S., Shahroz M., Zhang D., Kumar D. (2024). An Optimal Internet of Things-Driven Intelligent Decision-Making System for Real-Time Fishpond Water Quality Monitoring and Species Survival. Sensors.

[59] Marvin H.J.P., Van Asselt E., Kleter G., Meijer N., Lorentzen G., Johansen L.-H., Hannisdal R., Sele V., Bouzembrak Y. (2020). Expert-Driven Methodology to Assess and Predict the Effects of Drivers of Change on Vulnerabilities in a Food Supply Chain: Aquaculture of Atlantic Salmon in Norway as a Showcase. Trends Food Sci. Technol..

[60] Qian L., Yu G., Liu H., He Z. (2025). Segmentation and Calculation of Splashes Area during Fish Feeding Using Deep Learning and Linear Regression. Comput. Electron. Agric..

[61] Gkikas M.C., Gkikas D.C., Vonitsanos G., Theodorou J.A., Sioutas S. (2024). Application of Machine Learning for Predictive Analysis and Management of Mediterranean-Farmed Fish Mortalities: A Risk Management Case Study Using Apache Spark. Appl. Sci..

[62] Lara-Benítez P., Carranza-García M., Riquelme J.C. (2021). An Experimental Review on Deep Learning Architectures for Time Series Forecasting. Int. J. Neural Syst..

[63] Zhou X., Hao Y., Liu Y., Dang L., Qiao B., Zuo X. (2025). Short-Term Prediction of Dissolved Oxygen and Water Temperature Using Deep Learning with Dual Proportional-Integral-Derivative Error Corrector in Pond Culture. Eng. Appl. Artif. Intell..

[64] Liu Y., Zhang Q., Song L., Chen Y. (2019). Attention-Based Recurrent Neural Networks for Accurate Short-Term and Long-Term Dissolved Oxygen Prediction. Comput. Electron. Agric..

[65] Dewi T., Mardiyati E.N., Risma P., Oktarina Y. (2025). Hybrid Machine Learning Models for PV Output Prediction: Harnessing Random Forest and LSTM-RNN for Sustainable Energy Management in Aquaponic System. Energy Convers. Manag..

[66] Hadizadeh A., Tarokh M.J., Ghazani M.M. (2025). A Novel Transformer-Based Dual Attention Architecture for the Prediction of Financial Time Series. J. King Saud Univ. Comput. Inf. Sci..

[67] Zheng K., Yang R., Li R., Guo P., Yang L., Qin H. (2023). A Spatiotemporal Attention Network-Based Analysis of Golden Pompano School Feeding Behavior in an Aquaculture Vessel. Comput. Electron. Agric..

[68] Cui M., Zhao J., Mei X., Li D., Zeng L. (2026). Dual-Branch Mutual Learning for Multi-Modal to Single-Modal Fish Feeding Intensity Recognition in Aquaculture. Biosyst. Eng..

[69] Hasan M., Vasker N., Hossain M.M., Bhuiyan M.I., Biswas J., Ahmmad Rashid M.R. (2024). Framework for Fish Freshness Detection and Rotten Fish Removal in Bangladesh Using Mask R–CNN Method with Robotic Arm and Fisheye Analysis. J. Agric. Food Res..

[70] Li Y., Zhang L., He Y., Zhang X., Liu X. (2023). Intelligent Pulsed Ultraviolet c Radiation Sterilization System: A Cleaner Solution of Raw Ready-to-Eat Aquatic Products Processing. J. Clean. Prod..

[71] Lee P.G. (2000). Process Control and Artificial Intelligence Software for Aquaculture. Aquac. Eng..

[72] Zadeh L.A. (2015). Fuzzy Logic—A Personal Perspective. Fuzzy Sets Syst..

[73] Kudinov A.A., Kause A. (2024). Sex Identification in Rainbow Trout Using Genomic Information and Machine Learning. Genet. Sel. Evol..

[74] Li X., Yang B., Yang J., Fan Y., Qian X., Li H. (2021). Magnetic Properties and Its Application in the Prediction of Potentially Toxic Elements in Aquatic Products by Machine Learning. Sci. Total Environ..

[75] Wang P., Li Z., Cao L., Sui J., Lin H., Wang X., Wang K. (2025). Machine Learning-Assisted Early Evaluation of Aquatic Products Freshness Based on UV-Enhanced Multi-Enzymatic Cascade. Microchem. J..

[76] Yñiguez A.T., Ottong Z.J. (2020). Predicting Fish Kills and Toxic Blooms in an Intensive Mariculture Site in the Philippines Using a Machine Learning Model. Sci. Total Environ..

[77] Saberioon M., Císař P. (2018). Automated within Tank Fish Mass Estimation Using Infrared Reflection System. Comput. Electron. Agric..

[78] Han Q., Lu J., Zhu J., Lin L., Zheng Z., Jiang S. (2025). Non-Destructive Detection of Freshness in Crayfish (*Procambarus clarkii*) Based on near-Infrared Spectroscopy Combined with Deep Learning. Food Control.

[79] Wu Y., Wang X., Wang L., Zhang X., Shi Y., Jiang Y. (2023). Dynamic and Explainable Fish Mortality Prediction under Low-Concentration Ammonia Nitrogen Stress. Biosyst. Eng..

[80] Chen L., Yang X., Sun C., Wang Y., Xu D., Zhou C. (2020). Feed Intake Prediction Model for Group Fish Using the MEA-BP Neural Network in Intensive Aquaculture. Inf. Process. Agric..

[81] Zhang L., Wang J., Duan Q. (2020). Estimation for Fish Mass Using Image Analysis and Neural Network. Comput. Electron. Agric..

[82] Li W., Wu H., Zhu N., Jiang Y., Tan J., Guo Y. (2021). Prediction of Dissolved Oxygen in a Fishery Pond Based on Gated Recurrent Unit (GRU). Inf. Process. Agric..

[83] Shao K., Li D., Tang H., Zhang Y., Xu B., Bhatti U.A. (2025). Improving Multi-Step Dissolved Oxygen Prediction in Aquaculture Using Adaptive Temporal Convolution and Optimized Transformer. Comput. Electron. Agric..

[84] Mahmood F., Govindan R., Bermak A., Yang D., Khadra C., Al-Ansari T. (2021). Energy Utilization Assessment of a Semi-Closed Greenhouse Using Data-Driven Model Predictive Control. J. Clean. Prod..

[85] Kamali S., Ward V.C.A., Ricardez-Sandoval L. (2023). Closed-Loop Operation of a Simulated Recirculating Aquaculture System with an Integrated Application of Nonlinear Model Predictive Control and Moving Horizon Estimation. Comput. Electron. Agric..

[86] Soroush A., Giuffrè M., Chung S., Shung D.L. (2025). Generative Artificial Intelligence in Clinical Medicine and Impact on Gastroenterology. Gastroenterology.

[87] Fini C., Amato S.G., Scutaru D., Biancardi S., Antonucci F., Violino S., Ortenzi L., Nemmi E.N., Mei A., Pallottino F. (2025). Application of Generative Artificial Intelligence in the Aquacultural Sector. Aquac. Eng..

[88] Akram W., Din M.U., Saad Saoud L., Hussain I. (2026). A Review of Generative AI in Aquaculture: Applications, Case Studies and Challenges for Smart and Sustainable Farming. Aquac. Eng..

[89] Kim K., Myung H. (2018). Autoencoder-Combined Generative Adversarial Networks for Synthetic Image Data Generation and Detection of Jellyfish Swarm. IEEE Access.

[90] Yari Y., Næve I., Hammerdal A., Bergtun P.H., Måsøy S.-E., Voormolen M.M., Lovstakken L. (2024). Automated Measurement of Ovary Development in Atlantic Salmon Using Deep Learning. Ultrasound Med. Biol..

[91] Canovi N., Ellis B.A., Sørdalen T.K., Allken V., Halvorsen K.T., Malde K., Beyan C. (2024). Trajectory-Based Fish Event Classification through Pre-Training with Diffusion Models. Ecol. Inform..

[92] Treeprapin K., Bandatang R., Panthum T., Singchat W., Prasanpan J., Srikulnath K., Trirongjitmoah S. (2025). Enhancing Clariid Catfish Species Classification: A Deep Learning Framework Utilizing Cranial Morphology and Explainable AI. Smart Agric. Technol..

[93] Cai K., Yang Z., Gao T., Liang M., Liu P., Zhou S., Pang H., Liu Y. (2024). Efficient Recognition of Fish Feeding Behavior: A Novel Two-Stage Framework Pioneering Intelligent Aquaculture Strategies. Comput. Electron. Agric..

[94] Cook D., Middlemiss K., Jaksons P., Davison W., Jerrett A. (2019). Validation of Fish Length Estimations from a High Frequency Multi-Beam Sonar (ARIS) and Its Utilisation as a Field-Based Measurement Technique. Fish. Res..

[95] Adade S.Y.-S.S., Lin H., Johnson N.A.N., Qianqian S., Nunekpeku X., Ahmad W., Kwadzokpui B.A., Ekumah J.-N., Chen Q. (2024). Rapid Qualitative and Quantitative Analysis of Benzo(b)Fluoranthene (BbF) in Shrimp Using SERS-Based Sensor Coupled with Chemometric Models. Food Chem..

[96] Liu Z., Zhou C., Wan R., Xu L. (2023). Applying Artificial Intelligence to Optimize the Trawling Path and Operational Parameters for Antarctic Krill. Ocean Eng..

[97] Kunimatsu S., Kurota H., Muko S., Ohshimo S., Tomiyama T. (2025). Predicting Unseen Chub Mackerel Densities through Spatiotemporal Machine Learning: Indications of Potential Hyperdepletion in Catch-per-Unit-Effort Due to Fishing Ground Contraction. Ecol. Inform..

[98] Zhang T., Yang Y., Liu Y., Liu C., Zhao R., Li D., Shi C. (2024). Fully Automatic System for Fish Biomass Estimation Based on Deep Neural Network. Ecol. Inform..

[99] Tseng C.-H., Hsieh C.-L., Kuo Y.-F. (2020). Automatic Measurement of the Body Length of Harvested Fish Using Convolutional Neural Networks. Biosyst. Eng..

[100] Jang Y., Seo Y.-S. (2023). Machine Vision Based Fish Cutting Point Prediction for Target Weight. Comput. Mater. Contin..

[101] Zhang R., Chen X., Wan Z., Wang M., Xiao X. (2023). Deep Learning-Based Oyster Packaging System. Appl. Sci..

[102] Jia Z., Li M., Shi C., Zhang J., Yang X. (2022). Determination of Salmon Freshness by Computer Vision Based on Eye Color. Food Packag. Shelf Life.

[103] Sun Y., Zhang X. (2022). Tilapia Freshness Prediction Utilizing Gas Sensor Array System Combined with Convolutional Neural Network Pattern Recognition Model. Int. J. Food Prop..

[104] Liu H., You J., Liu C., Zhang Z., Sun A., Hao G., Shi X. (2024). Machine Learning-Assisted Wide-Gamut Fluorescence Visual Test Paper for Propazine Determination in Fish and Seawater Samples. Sens. Actuators B Chem..

[105] John E.P., Mishra U. (2024). Integrated Multitrophic Aquaculture Supply Chain Fish Traceability with Blockchain Technology, Valorisation of Fish Waste and Plastic Pollution Reduction by Seaweed Bioplastic: A Study in Tuna Fish Aquaculture Industry. J. Clean. Prod..

[106] Huang W., Wang Y., He W., Zhang X. (2025). RTI-Net: A Decision Support System for Fish Stress Classification Using Multimodal Learning Network. Expert Syst. Appl..

[107] Yang Y., Yu H., Zhang X., Zhang P., Tu W., Gu L. (2024). Fish Behavior Recognition Based on an Audio-Visual Multimodal Interactive Fusion Network. Aquac. Eng..

[108] Zhou C., Lin K., Xu D., Chen L., Guo Q., Sun C., Yang X. (2018). Near Infrared Computer Vision and Neuro-Fuzzy Model-Based Feeding Decision System for Fish in Aquaculture. Comput. Electron. Agric..

[109] Zhou Y., Zhang Q., Zhang H., Yang J., Guo Z., Bulugu I., Shen Y. (2023). A Deep Vision Sensing-based Fuzzy Control Scheme for Smart Feeding in the Industrial Recirculating Aquaculture Systems. Electron. Lett..

[110] Zhou X., Li D., Zhang L., Duan Q. (2021). Application of an Adaptive PID Controller Enhanced by a Differential Evolution Algorithm for Precise Control of Dissolved Oxygen in Recirculating Aquaculture Systems. Biosyst. Eng..

[111] Qiao S., Yu H., Song J., Zhang L., Zhao H., Huang W. (2025). TDA-GLM: Text Data Augmentation for Aquaculture Disease Prevention and Control via a Small Model-Guided ChatGLM. Aquac. Int..

[112] Danvirutai P., Charoenwattanasak S., Tola S., Thaiso K., Yuangsoi B., Minh H.T., Srichan C. (2025). An Integrating RAG-LLM and Deep Q-Network Framework for Intelligent Fish Control Systems. Sci. Rep..

[113] Brownscombe J.W., Hyder K., Potts W., Wilson K.L., Pope K.L., Danylchuk A.J., Cooke S.J., Clarke A., Arlinghaus R., Post J.R. (2019). The Future of Recreational Fisheries: Advances in Science, Monitoring, Management, and Practice. Fish. Res..

[114] Wang Y., Yu X., Liu J., An D., Wei Y. (2022). Dynamic Feeding Method for Aquaculture Fish Using Multi-Task Neural Network. Aquaculture.

[115] Marques J.C., Lackner S., Félix R., Orger M.B. (2018). Structure of the Zebrafish Locomotor Repertoire Revealed with Unsupervised Behavioral Clustering. Curr. Biol..

[116] Yang L., Liu Y., Yu H., Fang X., Song L., Li D., Chen Y. (2021). Computer Vision Models in Intelligent Aquaculture with Emphasis on Fish Detection and Behavior Analysis: A Review. Arch. Comput. Methods Eng..

[117] Zhao Z., Yang X., Zhao C., Zhou C. (2025). TMVF: Trusted Multi-View Fish Behavior Recognition with Correlative Feature and Adaptive Evidence Fusion. Inf. Fusion.

[118] Ahmed M.S., Aurpa T.T., Azad M.A.K. (2022). Fish Disease Detection Using Image Based Machine Learning Technique in Aquaculture. J. King Saud Univ.—Comput. Inf. Sci..

[119] Chung H., Lee J., Lee W.Y. (2021). A Review: Marine Bio-Logging of Animal Behaviour and Ocean Environments. Ocean Sci. J..

[120] Banan A., Nasiri A., Taheri-Garavand A. (2020). Deep Learning-Based Appearance Features Extraction for Automated Carp Species Identification. Aquac. Eng..

[121] Jose J.A., Sathish Kumar C., Sureshkumar S. (2022). A Deep Multi-Resolution Approach Using Learned Complex Wavelet Transform for Tuna Classification. J. King Saud Univ.—Comput. Inf. Sci..

[122] Salman A., Jalal A., Shafait F., Mian A., Shortis M., Seager J., Harvey E. (2016). Fish Species Classification in Unconstrained Underwater Environments Based on Deep Learning: Fish Classification Based on Deep Learning. Limnol. Oceanogr. Methods.

[123] Liu C., Wang Z., Li Y., Zhang Z., Li J., Xu C., Du R., Li D., Duan Q. (2023). Research Progress of Computer Vision Technology in Abnormal Fish Detection. Aquac. Eng..

[124] Ashraf Rather M., Ahmad I., Shah A., Ahmad Hajam Y., Amin A., Khursheed S., Ahmad I., Rasool S. (2024). Exploring Opportunities of Artificial Intelligence in Aquaculture to Meet Increasing Food Demand. Food Chem. X.

[125] Xie X., Zhang B., Wang X., Jiang Y., Buchmann K., Zhou S., Li Y., Yin F., Galindo-Villegas J. (2025). A Machine Learning-Driven Early Warning System for Cryptocaryoniasis in Marine Aquaculture. Parasites Vectors.

[126] Liu W., Lyu J., Wu D., Cao Y., Ma Q., Lu Y., Zhang X. (2022). Cutting Techniques in the Fish Industry: A Critical Review. Foods.

[127] Li Q., Ma H., Min W., Wang Y., Zhao R., Zhou Y., Tan Y., Luo Y., Hong H. (2024). Recent Advances in Fish Cutting: From Cutting Schemes to Automatic Technologies and Internet of Things Innovations. Compr. Rev. Food Sci. Food Saf..

[128] Khodabandehloo K. (2022). Achieving Robotic Meat Cutting. Anim. Front..

[129] Azarmdel H., Mohtasebi S.S., Jafary A., Behfar H., Rosado Muñoz A. (2021). Design and Simulation of a Vision-Based Automatic Trout Fish-Processing Robot. Appl. Sci..

[130] Hassoun A., Boukid F., Ozogul F., Aït-Kaddour A., Soriano J.M., Lorenzo J.M., Perestrelo R., Galanakis C.M., Bono G., Bouyahya A. (2023). Creating New Opportunities for Sustainable Food Packaging through Dimensions of Industry 4.0: New Insights into the Food Waste Perspective. Trends Food Sci. Technol..

[131] Jyoti, Gupta A.K., Kumar A., Kumar B. (2025). Advancing Sustainable Food Packaging: Integrating Machine Learning, Deep Learning, and Artificial Intelligence. Trends Food Sci. Technol..

[132] Paradkar V., Raheman H., K. R. (2021). Development of a Metering Mechanism with Serial Robotic Arm for Handling Paper Pot Seedlings in a Vegetable Transplanter. Artif. Intell. Agric..

[133] Lv Z., Chen T., Cai Z., Chen Z. (2023). Machine Learning-Based Garbage Detection and 3D Spatial Localization for Intelligent Robotic Grasp. Appl. Sci..

[134] Jiao X., Zhu J., Ye W., Zou H., Yan B., Zhang N., Qiang J., Tao Y., Zhang H., Zhang D. (2025). Artificial Intelligence in Smart Seafood Safety across the Supply Chains: Recent Advances and Future Prospects. Trends Food Sci. Technol..

[135] Moosavi-Nasab M., Khoshnoudi-Nia S., Azimifar Z., Kamyab S. (2021). Evaluation of the Total Volatile Basic Nitrogen (TVB-N) Content in Fish Fillets Using Hyperspectral Imaging Coupled with Deep Learning Neural Network and Meta-Analysis. Sci. Rep..

[136] Wang K., Yue Z., Lin H., Wang Q., Wang L., Tian Y., Ren L. (2023). Rapid Classification of the Freshness Grades of Sea Bass (*Lateolabrax japonicus*) Fillets Using a Portable Raman Spectrometer with Machine Learning Method. Microchem. J..

[137] You J., Sun Z., Li X., Ying X., Shi C., Fan H. (2024). Noninvasive Freshness Evaluation of Bighead Carp Heads Based on Fluorescence Spectroscopy Coupled with Long Short-Term Memory Network: Simulation of Cold Chains. Food Innov. Adv..

[138] Wijaya D.R., Syarwan N.F., Nugraha M.A., Ananda D., Fahrudin T., Handayani R. (2023). Seafood Quality Detection Using Electronic Nose and Machine Learning Algorithms with Hyperparameter Optimization. IEEE Access.

[139] Zhang L., Zhang M., Mujumdar A.S., Wang D. (2024). Deep Learning Used with a Colorimetric Sensor Array to Detect Indole for Nondestructive Monitoring of Shrimp Freshness. ACS Appl. Mater. Interfaces.

[140] Jiao Z., Yu N., Mao J., Yang Q., Jiao L., Wang X., Shi W., Yu H., Wei S. (2023). The Occurrence, Tissue Distribution, and PBT Potential of per- and Polyfluoroalkyl Substances in the Freshwater Organisms from the Yangtze River via Nontarget Analysis. J. Hazard. Mater..

[141] Bittsánszky A., Bilicki V., Sudár G., Süth M., Kusza S., Tóth A.J. (2026). Artificial Intelligence in Postharvest Food Safety Control of Animal-Source Foods: Evidence Thresholds, Validation, and Regulatory Applicability. Vet. Sci..

[142] Probst W.N. (2020). How Emerging Data Technologies Can Increase Trust and Transparency in Fisheries. ICES J. Mar. Sci..

[143] Alsharabi N., Ktari J., Frikha T., Alayba A., Alzahrani A.J., Jadi A., Hamam H. (2024). Using Blockchain and AI Technologies for Sustainable, Biodiverse, and Transparent Fisheries of the Future. J. Cloud Comp..

[144] Tsolakis N., Schumacher R., Dora M., Kumar M. (2023). Artificial Intelligence and Blockchain Implementation in Supply Chains: A Pathway to Sustainability and Data Monetisation?. Ann. Oper. Res..

[145] Hassoun A., Aït-Kaddour A., Abu-Mahfouz A.M., Rathod N.B., Bader F., Barba F.J., Biancolillo A., Cropotova J., Galanakis C.M., Jambrak A.R. (2023). The Fourth Industrial Revolution in the Food Industry—Part I: Industry 4.0 Technologies. Crit. Rev. Food Sci. Nutr..

